# Supplementary material for: Fine-tuning a local LLaMA-3 large language model for automated privacy-preserving physician letter generation in radiation oncology
Source: Front Artif Intell. 2025 Jan 14;7:1493716. doi: 10.3389/frai.2024.1493716 (PMC11772293; doi:10.3389/frai.2024.1493716)
Supplement: Supplementary file 1 [file Data_Sheet_1.pdf]

Input

Diagnoses
Low intermediate risk adenocarcinoma of the prostate cT1c cN0 cM0 iPSA 5.36 ng/ml Gleason score 3+4=7a (ED 01/2019)

Secondary diagnoses
History of epididymal carcinoma 1975
History of Partial thyroid resection for nodular goiter
TVT right leg 12/2008 and knee area 80s
Slightly restricted left ventricular pump function, most likely chemotoxic cardiomyopathy
Renal insufficiency in the stage of compensated retention
Colon carcinoma, history after 3x operations and chemotherapy

Tumor-specific anamnesis
04/2005: Extended right hemicolectomy for adenocarcinoma of the transverse colon, pT3 pN2 cM0 G2 R0.
05- 10/2005: 6 cycles of adjuvant chemotherapy according to the Majo protocol.
11/2005: Anterior rectal resection for denovo-adenocarcinoma of the rectosigmoid colon, pT1 pN0 cM0 G2 R0. 06/2008: Control colonoscopy. Polyp removal and histological evidence of a new adenocarcinoma in the remaining colon (high-grade intraepithelial neoplasia with questionable invasion into the muscularis propria).
07/2008: Exploratory laparotomy, remaining proctocolectomy with ileoanal pouch creation and protective double-barreled ileostomy.
09/2008-02/2009: Cycles 1-10 of adjuvant chemotherapy according to the FOLFOX-4 regimen. Complication: Increasing PNP and diarrhea under oxaliplatin.
02- 03/2009: 4 cycles of monotherapy with 5-FU/FA.
03/2009: Completion of adjuvant therapy. Conclusion: No evidence of disease (NED).
01/2019: Intermediate risk prostate cancer cT1c cNx cMx Gleason score 3+4=7a iPSA 5.36 ng/ml Presentation at the Martini Clinic in Hamburg and Heidelberg University Hospital for radiotherapy treatment or tumor resection.

Recommendation: Due to previous tumor diseases and surgical treatments for colon cancer, interstitial brachytherapy was recommended.
28.02.2019: Presentation in Urology Tumor Board Recommendation: Brachytherapy
19.03.2019: Interstitial brachytherapy: 1st series: HDR brachytherapy:
18.04.2019: Interstitial brachytherapy: 2nd series: HDR brachytherapy:

Course
The interstitial brachytherapy was able to be applied in a timely manner at the full dosage. The procedure and the removal of the applicators were uncomplicated; no significant side effects occurred. The implantation sites were free of irritation upon discharge; there was no secondary bleeding. The patient was discharged in a stable general condition.

Assessment
Procedure: 1) Please present yourself for the 3rd session in the radiation surgery on May 27, 2019 at 8:00 a.m. 2) Tumor-appropriate follow-up examination via your treating urologist; please make appointments yourself. 3) A first radiotherapy follow-up appointment in our outpatient clinic was scheduled for August 8, 2019 at 11:40 a.m.
