# Supplementary material for: Fine-tuning a local LLaMA-3 large language model for automated privacy-preserving physician letter generation in radiation oncology
Source: Front Artif Intell. 2025 Jan 14;7:1493716. doi: 10.3389/frai.2024.1493716 (PMC11772293; doi:10.3389/frai.2024.1493716)
Supplement: Supplementary file 2 [file Data_Sheet_2.pdf]

Here is an example of the question-answer (input-and-output) style for fine-tuning.

{"input": "Diagnosis Diagnosis: Glioblastoma left temporo-occipital, IDH-1 wild type, WHO grade IV, MGMT promoter methylation Secondary diagnoses: Art. Hypertension Pollen allergy Tumor-specific anamnesis ##.##.####: Left temporo-parietal, basal craniotomy Tumor resection (Neurosurgical Clinic, University of Erlangen) Histology (Neuropathological Institute, University of Erlangen): Glioblastoma, IDH-1 wild type, WHO grade IV. ##.##.####: MRI skull assessment: No evidence of high-grade tumor residue/recurrence after extirpation of a glioblastoma multiforme WHO grade IV temporoparietal left on December 2nd ####. Postoperative changes, or low-grade tumor parts adjacent to the resection defect. ##.##.#### - ##.##.####: Postoperative simultaneous Radiochemotherapy Radiotherapy in VMAT Single dose 2 Gy, total dose 60 Gy Chemotherapy CeCeNu 100 mg/m^2 BSA d 1, 200 mg absolute Temodal 100 mg/m^2 BSA d 2-6, 200 mg absolute WH d 42 to #/#### Maintenance chemotherapy with CCNU/Temodal (total of 6 cycles, no Temodal in cycle 6) [...]

"output": "The patient was diagnosed with a left temporoparietal glioblastoma, WHO Grade IV, with IDH-1 wild type and MGMT promoter methylation. On December #, ####, he underwent a basal craniotomy for tumor resection, which confirmed the diagnosis of glioblastoma. Subsequent MRI on January ##, ####, showed no evidence of higher-grade residual tumor after the resection, although there were postoperative changes and low-grade tumor remnants adjacent to the resection defect.\n\nFollowing the surgery, the patient received concurrent radiochemotherapy from January ##, ####, to March #, ####, with radiotherapy (60 Gy) and chemotherapy (CeCeNu and Temodal). He is currently undergoing maintenance chemotherapy with CCNU/Temodal, with the next follow-up and interdisciplinary tumor center coordination planned according to the guidelines for radiation protection in medicine. [...]" },
