# Supplementary material for: Fine-tuning a local LLaMA-3 large language model for automated privacy-preserving physician letter generation in radiation oncology
Source: Front Artif Intell. 2025 Jan 14;7:1493716. doi: 10.3389/frai.2024.1493716 (PMC11772293; doi:10.3389/frai.2024.1493716)
Supplement: Supplementary file 3 [file Data_Sheet_3.pdf]

German

Modell-Input 1:

Dokument Abgeschlossen am: 18.02.2019
Abgeschlossen von: Dr. med. Godehard Lahmer

Briefkopf ST-HSA
Ansprechpartner: Dr. Godehard Lahmer

Betreff:
Herrn **** ****, geb. **.**.****
9403* Passau

Diagnosen:
Hypophysenadenom mit immunhistochemisch nachweisbarer Expression von Prolaktin (laktotropes Adenom) STH, ACTH, TSH, FSH, LH neg. Prolaktin mittel ++++

Nebendiagnosen:
Diabetes mellitus II
Art. Hypertonus
Depression
Blepharochalasis bds.
Gesichtsfelddefekte links
Myopie links
Astigmatismus links
Presbyopie bds.

Tumorspezifische Anamnese:
2016: Quinagolid + Cabergolin.
19.09.2018 Transsphenoidale Operation (Neurochirurgische Klinik, Universität Erlangen).
Histologie (Neuropathologisches Institut, Universität Erlangen):
Hypophysenadenom mit immunhistochemisch nachweisbarer Expression von Prolaktin (laktotropes Adenom).
STH, ACTH, TSH, FSH, LH neg.
Prolaktin mittel ++++

27.09.2018: Tumorboard ZNS-Tumore, Universität Erlangen:
Verlaufskontrolle in 3 Monaten mit MRT und je nach Ergebnis gegebenenfalls Bestrahlung.

Empfehlung:
Fraktioniert stereotaktische Strahlentherapie (wenn möglich im Rahmen der HippoSpare-01-Studie).

Empfänger:
Prof. Dr. med. Michael Buchfelder, Direktor der Neurochirurgischen Klinik, Schwabachanlage 6, 91054 Erlangen
N: Dr. med. Alois Schätzl, Arzt für Allgemeinmedizin, Ebersberger Straße 16, 94113 Tiefenbach
N: Dr. med. Birgit Großer, Fachärztin für Innere Medizin, Stadtgraben 1, 94315 Straubing

English

Model input 1:

Document Completed on: 18.02.2019
Completed by: Dr. med. Godehard Lahmer

Subject:
Mr. **** ***, born **.**.****
9403* Passau

Diagnoses:
Pituitary adenoma with immunohistochemically detectable expression of prolactin (lactotropic adenoma) STH, ACTH, TSH, FSH, LH neg. Prolactin expression medium ++++

Secondary Diagnoses:
Diabetes mellitus II
Art. Hypertension
Depression
Blepharochalasis on both sides Visual field defects on the left
Myopia on the left
Astigmatism on the left
Presbyopia on both sides

Tumor-specific history:
2016: Quinagolide + cabergoline.

17.08.2018: Transsphenoidal surgery (Neurosurgical Clinic, University of Erlangen). Histology (Neuropathic Institute, University of Erlangen): Pituitary adenoma with immunohistochemically detectable expression of prolactin (lactotropic adenoma). STH, ACTH, TSH, FSH, LH neg. Prolactin medium ++++

24.08.2018: Tumor board CNS tumors, University of Erlangen: Follow-up in 3 months with MRI and, depending on the result, radiation if necessary.

Recommended treatment:
Fractionated stereotactic radiotherapy (if possible as part of the HippoSpare-01 study).

Recipients:
Prof. Dr. med. Michael Buchfelder, Director of the Neurosurgical Clinic, Schwabachanlage 6, 91054 Erlangen
N: Dr. med. Alois Schätzl, General Practitioner, Ebersberger Straße 16, 94113 Tiefenbach
N: Dr. med. Birgit Großer, Specialist in Internal Medicine, Stadtgraben 1, 94315 Straubing

German

KI-Brief – Fall 1:

Sehr geehrter Herr Professor Buchfelder,

wir berichten Ihnen über unseren gemeinsamen Patienten, Herrn **** ****, der sich am 15.02.2019 zu einem Aufklärungsgespräch in unserer strahlentherapeutischen Ambulanz vorstellte.

Der Patient wurde an Ihrer Klinik am 19.09.2018 transsphenoidal operiert. Histopathologisch zeigte sich ein Prolaktinproduzierendes Hypophysenadenom.

Wir haben den Patienten nun über eine fraktioniert stereotaktische Strahlentherapie aufgeklärt und beraten.
Der Patient sagte zum Abschluss des Gespräches, dass er sich noch nicht für die Durchführung einer Strahlentherapie entscheiden könne, sondern zunächst noch eine erneute Verlaufskontrolle im Sommer 2019 wahrnehmen möchte.

Sollte sich der Patient für die Durchführung einer Strahlentherapie entscheiden, dann würde er sich selbständig wieder in unserer strahlentherapeutischen Ambulanz melden.

Wir bedanken uns für die kollegiale Zusammenarbeit und verbleiben

mit freundlichen kollegialen Grüßen

Prof. Dr. R. Fietkau
Direktor der Klinik

Dr. G. Lahmer
Oberarzt der Klinik

#	Mache ein Kreuzchen für jede Kategorie!	✘

Korrektheit: Bitte nicht die Datumsangaben bewerten, die nicht in den Inputdaten enthalten sind, diese sind eine Schätzung der KI aufgrund der Datumsangaben und des Zeitpunkts, an dem der Brief abgeschlossen wurde.

1	Schwerwiegende Fehler, Risiko für klin. Fehlentscheidungen	
2	Relevante Fehler, ohne klinische Auswirkung	
3	Kleinere Ungenauigkeiten, irrelevant für Patientenfall	
4	Der Brief enthält keine Fehler	

Notwendigkeit für Anpassungen:

1	Der Brief ist so unvollständig, dass es schneller ist, den Brief neu zu schreiben	
2	Der Brief benötigt größere Anpassungen > 1 min	
3	Der Brief benötigt kleiner Anpassungen <= 1 min	
4	Der Brief ist vollständig und benötigt keine Anpassungen	

Klinik- / Institutsspezifische Inhalte und Stil – Brief:

1	Keinerlei klinikspezifische Inhalte oder Anpassung an den lokalen Briefstil	
2	In sehr geringem Maße klinikspezifische Inhalte oder Anpassung an den lokalen Stil	

3	Der Brief enthält erhebliche klinischspezifische Inhalte oder Anpassungen an den lokalen Stil	
4	Der Brief bildet vollständig den Stil eines lokalen Briefes ab	

Einschätzung Praktischer Nutzen – Brief:

1	Kein Nutzen für die Brieferstellung	
2	Kleiner Nutzen für die Brieferstellung	
3	Mäßiger Nutzen für die Brieferstellung	
4	Großer Nutzen für die Brieferstellung	

English

AI Letter – Case 1:

Dear Professor Buchfelder,

We are reporting to you about our mutual patient, Mr. ***** ******, who came to our radiotherapy outpatient clinic for an information session on 15.02.2019.

The patient underwent transsphenoidal surgery at your clinic on 17.08.2018. Histopathology revealed a prolactin-producing pituitary adenoma.

We have now informed and advised the patient about fractionated stereotactic radiotherapy. At the end of the conversation, the patient said that he could not yet decide whether to undergo radiotherapy, but would first like to have another follow-up check in summer 2019. If the patient decides to undergo radiotherapy, he would contact our radiotherapy outpatient clinic again on his own.

We would like to thank you for your friendly cooperation and remain
with kind regards

Prof. Dr. R. Fietkau
Director of the clinic

Dr. G. Lahmer
Senior Physician of the Clinic

#	**Make a mark for each category!**	✘

Correctness: Please do not rate the date details that are not included in the input data; these are an estimate by the AI based on the dates and the time when the letter was completed.

1	Severe errors, risk for clinical misdecisions	
2	Relevant errors, no clinical impact	
3	Minor inaccuracies, irrelevant to the patient case	
4	The letter contains no errors	

Need for Adjustments:

1	The letter is so incomplete that it is faster to rewrite the letter	
2	The letter requires major adjustments > 1 min	
3	The letter requires minor adjustments <= 1 min	
4	The letter is complete and requires no adjustments	

Clinic-/Institute-Specific Content and Style – Letter:

1	No clinic-specific content or adjustment to local letter style	
2	Very little clinic-specific content or adjustment to local style	
3	The letter contains significant clinic-specific content or adjustments to the local style	
4	The letter fully reflects the style of a local letter	

Assessment Practical Utility – Letter:

1	No benefit for letter creation	
2	Small benefit for letter creation	

3	Moderate benefit for letter creation	
4	Great benefit for letter creation	

German

Original-Brief (Arzt) – Fall 1:

Sehr geehrter Herr Professor Buchfelder,

Wir berichten Ihnen über unseren gemeinsamen Patienten, Herrn **** ****, der sich am 15.02.2019 zu einem Aufklärungsgespräch in unserer strahlentherapeutischen Ambulanz vorstellte.

An Ihrer Klinik erfolgte am 17.09.2018 eine transsphenoidale Operation. Histopathologisch zeigte sich ein Hypophysenadenom (lactotropes Adenom).

Nun haben wir ihn über die Möglichkeiten einer fraktioniert stereotaktischen Strahlentherapie beraten. Der Patient sagte zum Abschluss des Gespräches, dass er sich noch nicht für die Durchführung einer Strahlentherapie entscheiden könne, sondern noch etwas Bedenkzeit braucht.

Sollte sich der Patient für die Durchführung einer Strahlentherapie entscheiden, dann würde er sich selbständig in unserer strahlentherapeutischen Klinik melden. Ebenso würden wir dann im weiteren Verlauf alles Weitere koordinieren.

Wir bedanken uns für die kollegiale Zusammenarbeit und verbleiben

mit freundlichen kollegialen Grüßen

Prof. Dr. R. Fietkau
Direktor der Klinik

Dr. G. Lahmer
Oberarzt der Klinik

#	Mache ein Kreuzchen für jede Kategorie!	✘

Vergleich KI-Brief mit dem Originalbrief:

1	Der Originalbrief ist besser	
2	Originalbrief und KI-Brief sind gleich gut	
3	Der KI-Brief ist besser	

English

Original Letter (Doctor) – Case 1:

Dear Professor Buchfelder,

We are reporting to you about our mutual patient, Mr. **** ****, who came to our radiotherapy outpatient clinic for an information session on 15.02.2019.

A transsphenoidal operation was performed at your clinic on 17.08.2018. Histopathology revealed a pituitary adenoma (lactotropic adenoma).

Now we have advised him about the possibilities of fractionated stereotactic radiotherapy. At the end of the conversation, the patient said that he could not yet decide whether to undergo radiotherapy, but needed some time to think about it. If the patient decides to undergo radiotherapy, he would contact our radiotherapy clinic on his own. We would also coordinate everything else in the further course of events.

We would like to thank you for your friendly cooperation and remain
with kind regards,

Prof. Dr. R. Fietkau
Director of the clinic

Dr. G. Lahmer
Senior Physician of the Clinic

#	**Make a mark for each category!**	✘

Comparison of AI letter with the original letter:

1	The original letter is better	
2	The original letter and the AI letter are equally good	
3	The AI letter is better	

German

Modell-Input 2:

Dokument Abgeschlossen am: 08.10.2021
Abgeschlossen von: Dr. med. Godehard Lahmer

Briefkopf ST-PRI
Ansprechpartner: Dr. Godehard Lahmer

Betreff
Herrn **** ****, geb. **.**.****
904** Nürnberg

Diagnosen:
Diagnose: Prostatakarzinom
cT2a cN0 cM0
Gleason-Score 3+3=6 iPSA 7,61 ng/ml
Nebendiagnosen:
Allergien: Latex, Lidocain
Adipositas
Depression
Angststörung
Art. Hypertonus

Tumorspezifische Anamnese:
04.04.2014:
MRT-Stanzbiopsie der Prostata (Uni-Klinik Erlangen).
Histologie: Man sieht hier in 2/6 Stanzen links, Basis medial links und Mitte lateral links eine bis max. 50%-ige Infiltration (max. 5 mm) durch ein mäßig differenziertes Prostatakarzinom (Gleason-Score 6, 3+3). Die sieben Stanzbiopsien rechts tumorfrei.
09.2014:
HIFU-Hemiablation links.
02.2016:
HIFU-Hemiablation links.
23.04.2021:
PSMA-PET-CT:
Keine Voraufnahmen zum Vergleich vorliegend. Bei mittlerweile zweimaliger Ablation der Prostata links hochgradiger Verdacht auf ein Lokalrezidiv links posterolateral basisnah (6p,7p). Darüberhinaus keine anderweitige, insbesondere lymphogene oder ossäre Manifestation.
Nebenbefundlich:
Steatosis hepatis. Deutliche Degenerationen der Wirbelsäule. Koxarthrose, Gonarthrose. Blande Leberzyste Segment 4a.
Empfehlung: Alleinige interstitielle Brachytherapie, z.B. als permanente Brachytherapie mit Jod-Seeds.

Empfänger:
N: Priv.Doz. Dr. Bastian Keck, Goethestr. 54, 91054 Erlangen
N: Dr. med. Christian Renard, Lausitzer Str. 4, 90453 Nürnberg

English

<u>Model input 2:</u>

Document Completed on: 08.10.2021
Completed by: Dr. med. Godehard Lahmer

Letterhead ST-PRI
Contact Dr. med. S. Godehard Lahmer

Subject Mr. **** ****, born **.**.****
904** Nuremberg

Diagnosis:
Diagnosis: Prostate Cancer
cT2a cN0 cM0
Gleason Score 3+3=6, iPSA 7.61 ng/ml

Secondary diagnoses:
Allergies: Latex, Lidocaine
Obesity
Depression
Anxiety Disorder
Arterial Hypertension

Tumor-specific history:
04.04.2014:
MRI-guided prostate biopsy (University Hospital Erlangen). Histology: Two of six biopsies on the left side, medial base left, and lateral mid left, show up to 50% infiltration (max 5 mm) by a moderately differentiated prostate carcinoma (Gleason Score 6, 3+3). The seven biopsies on the right side were tumor-free.
09.2014:
HIFU-Hemiablation left side.
02.2016:
HIFU-Hemiablation left side.
23.04.2021:
PSMA-PET-CT: No prior images available for comparison. After two ablations of the left side of the prostate, there is a high suspicion of a local recurrence on the left posterolateral base (6p,7p). Additionally, no other lymphatic or osseous manifestations are present. Incidental findings: Hepatic steatosis. Significant degeneration of the spine. Coxarthrosis, gonarthrosis. Bland liver cyst segment 4a.

Recommendation: Interstitial brachytherapy alone, e.g. as permanent brachytherapy with iodine seeds.

Recipient:
N: Priv.Doz. Dr. Bastian Keck, Goethestr. 54, 91054 Erlangen
N: Dr. med. Christian Renard, Lausitzer Str. 4, 90453 Nürnberg

German:

KI-Brief – Fall 2:

Sehr geehrter Herr PD Dr. Keck,

wir berichten Ihnen über unseren gemeinsamen Patienten, Herrn **** ****, der sich am 08.10.2021 in unserer strahlentherapeutischen Privatambulanz zu einem Beratungsgespräch vorstellte.
Die tumorspezifische Anamnese ist Ihnen selbst bestens bekannt und dem Brief oberhalb kurz skizziert zu entnehmen.

Bei Prostatakarzinom und Z. n. zweimaliger HIFU-Therapie der Prostata links, zeigt sich nun bildgebend der hohe Verdacht auf ein Lokalrezidiv links posterolateral.

Wir haben mit dem Patienten über potentielle Therapiemethoden gesprochen (Operation oder Strahlentherapie).
Insbesondere haben wir ihm die Möglichkeiten der Strahlentherapie erklärt und ihm insbesondere die Durchführung einer alleinigen interstitiellen Brachytherapie empfohlen.

Der Patient sagte zum Abschluss des Gespräches, dass er nun noch etwas Bedenkzeit braucht. Er würde sich selbstständig melden, sofern er eine Strahlentherapie wünscht.

Mit freundlichen kollegialen Grüßen

Prof. Dr. R. Fietkau
Direktor der Klinik

Dr. G. Lahmer
Oberarzt der Klinik

#	Mache ein Kreuzchen für jede Kategorie!	✘

Korrektheit: Bitte nicht die Datumsangaben bewerten, die nicht in den Inputdaten enthalten sind, diese sind eine Schätzung der KI aufgrund der Datumsangaben und des Zeitpunkts, an dem der Brief abgeschlossen wurde.

1	Schwerwiegende Fehler, Risiko für klin. Fehlentscheidungen	
2	Relevante Fehler, ohne klinische Auswirkung	
3	Kleinere Ungenauigkeiten, irrelevant für Patientenfall	
4	Der Brief enthält keine Fehler	

Notwendigkeit für Anpassungen:

1	Der Brief ist so unvollständig, dass es schneller ist, den Brief neu zu schreiben	
2	Der Brief benötigt größere Anpassungen > 1 min	
3	Der Brief benötigt kleiner Anpassungen <= 1 min	
4	Der Brief ist vollständig und benötigt keine Anpassungen	

Klinik- / Institutsspezifische Inhalte und Stil – Brief:

1	Keinerlei klinikspezifische Inhalte oder Anpassung an den lokalen Briefstil	
2	In sehr geringem Maße klinikspezifische Inhalte oder Anpassung an den lokalen Stil	

3	Der Brief enthält erhebliche klinischspezifische Inhalte oder Anpassungen an den lokalen Stil	
4	Der Brief bildet vollständig den Stil eines lokalen Briefes ab	

Einschätzung Praktischer Nutzen – Brief:

1	Kein Nutzen für die Brieferstellung	
2	Kleiner Nutzen für die Brieferstellung	
3	Mäßiger Nutzen für die Brieferstellung	
4	Großer Nutzen für die Brieferstellung	

English

<u>AI Letter – Case 2:</u>

We are reporting on our mutual patient, Mr. **** **** who presented himself on 08.10.2021 in our private radiation therapy outpatient clinic for a consultation. The tumor-specific history is already well known to you and is briefly outlined in the letter above.

In the case of prostate cancer and history after two HIFU treatments of the left prostate, imaging now shows a high suspicion of a local recurrence on the left posterolateral side.

We spoke to the patient about potential treatment methods (surgery or radiotherapy). In particular, we explained the options for radiotherapy to him and recommended interstitial brachytherapy alone.

At the end of the conversation, the patient said that he now needed some time to think about it. He would contact us himself if he wanted radiotherapy.

With kind collegial regards,

Prof. Dr. R. Fietkau
Director of the clinic

Dr. G. Lahmer
Senior Physician of the Clinic

#	Make a mark for each category!	✘

Correctness: Please do not rate the date details that are not included in the input data; these are an estimate by the AI based on the dates and the time when the letter was completed.

1	Severe errors, risk for clinical misdecisions	
2	Relevant errors, no clinical impact	
3	Minor inaccuracies, irrelevant to the patient case	
4	The letter contains no errors	

Need for Adjustments:

1	The letter is so incomplete that it is faster to rewrite the letter	
2	The letter requires major adjustments > 1 min	
3	The letter requires minor adjustments <= 1 min	
4	The letter is complete and requires no adjustments	

Clinic-/Institute-Specific Content and Style – Letter:

1	No clinic-specific content or adjustment to local letter style	
2	Very little clinic-specific content or adjustment to local style	
3	The letter contains significant clinic-specific content or adjustments to the local style	
4	The letter fully reflects the style of a local letter	

Assessment Practical Utility – Letter:

1	No benefit for letter creation	
2	Small benefit for letter creation	
3	Moderate benefit for letter creation	
4	Great benefit for letter creation	

German

Original-Brief (Arzt) – Fall 2:

Sehr geehrter Herr PD Dr. Keck,

wir berichten Ihnen über unseren gemeinsamen Patienten, Herrn **** ****, der sich am 13.06.2021 in unserer strahlentherapeutischen Privatambulanz zu einem Aufklärungsgespräch vorstellte.

Bei low-risk Prostatakarzinom und Z. n. 2x HIFU haben wir nun mit dem Patienten bei persistierendem PSA-Wert von ca. 7 ng/ml über potentielle strahlentherapeutische Methoden gesprochen (perkutane Strahlentherapie, kombinierte perkutane Strahlentherapie mit Brachytherapie-Boost, alleinige permanente Brachytherapie mit Jod-Seeds, temporäre Brachytherapie mit Iridium-192, PDR-Verfahren, HDR-Verfahren). Der Patient tendiert zu einer Behandlung mittels Jod-Seeds. Er sagte zum Abschluss des Gespräches, dass er im Falle einer Strahlentherapie diese gerne im Frühjahr 2022 durchführen lassen möchte und würde sich selbstständig melden für eine weitere Terminkoordinierung.

Wir bedanken uns für die kollegiale Zusammenarbeit und verbleiben

mit freundlichen kollegialen Grüßen

Prof. Dr. R. Fietkau
Direktor der Klinik

Dr. G. Lahmer
Oberarzt der Klinik

#	**Mache ein Kreuzchen für jede Kategorie!**	✘

Vergleich KI-Brief mit dem Originalbrief:

1	Der Originalbrief ist besser	
2	Originalbrief und KI-Brief sind gleich gut	
3	Der KI-Brief ist besser	

English

Original Letter (Doctor) – Case 2:

We are reporting on our mutual patient, Mr. **** **** who presented himself on 13.06.2021 at our private radiation therapy outpatient clinic for an informational consultation.

In the case of low-risk prostate cancer and history after 2x HIFU, we have now spoken to the patient with a persistent PSA value of approx. 7 ng/ml about potential radiotherapy methods (percutaneous radiotherapy, combined percutaneous radiotherapy with brachytherapy boost, permanent brachytherapy with iodine seeds alone, temporary brachytherapy with iridium-192, PDR procedure, HDR procedure). The patient is leaning towards treatment with iodine seeds. At the end of the conversation, he said that if he wanted radiotherapy, he would like to have it done in spring 2022 and would contact him himself to coordinate further appointments.

We thank you for the collegial cooperation and remain

with kind collegial regards,

Prof. Dr. R. Fietkau
Director of the clinic

Dr. G. Lahmer
Senior Physician of the Clinic

#	Make a mark for each category!	✘

Comparison of AI letter with the original letter:

1	The original letter is better	
2	The original letter and the AI letter are equally good	
3	The AI letter is better	

German

Modell-Input 3:

Dokument Abgeschlossen am: 28.02.2018
Abgeschlossen von: Dr. med. Godehard Lahmer

Briefkopf ST-HSA
Ansprechpartner Dr. Godehard Lahmer

Betreff
Frau **** ****, geb. **.**.****
917** ***

Diagnose:
1. Glomus jugulare Tumor rechts (Typ C1 nach Fisch und Valavanis)
2. Z.n. Kolonkarzinom (ED 1995)

Nebendiagnosen:
Hyperlipoproteinämie
Z.n. Appendektomie

Tumorspezifische Anamnese:
08.01.2018:
Felsenbein-MRT (Radiologisches Institut, Universität Erlangen):
Befund vereinbar mit Glomus jugulare Tumor rechts (Typ C1 nach Fisch und Valavanis).

19.02.2018:
Teil-Entfernung Glomus jugulare-Tumor rechts (HNO-Klinik, Universität Erlangen).
Histologie (E.-Nr.: ****, Pathologisches Institut, Universität Erlangen):
Paragangliom des Glomus tympanicum (sog. Glomustumor). Kein Verlust von SDHB.

21.02.2018:
Tumorboardempfehlung Kopf-/Hals-Tumore, Universität Erlangen:
Radiatio des Resttumors.
Geplant: Fraktioniert stereotaktische Strahlentherapie im Bereich des Resttumors bei Glomus jugulare Tumor rechts.

Empfänger
Prof. Dr. med. Dr. h.c. Heinrich Iro, Direktor der HNO-Klinik, Waldstraße 1, 91054 Erlangen
N: Dr. med. Martin Weichmann, HNO-Arzt, Marktplatz 8, 91781 Weißenburg

English

Model input 3:

Document Completed on: 28.02.2018
Completed by: Dr. med. Godehard Lahmer

Letterhead ST-HAS
Contact Dr. med. Godehard Lahmer

Subject
Ms. ****, born ..
917** ***

Diagnosis:
Glomus jugulare tumor right side (Type C1 according to Fisch and Valavanis)
History of colon cancer (initial diagnosis in 1995)
Secondary Diagnoses:
Hyperlipoproteinemia
History of appendectomy

Tumor-specific history:
08.01.2018:
Temporal bone MRI (Radiological Institute, University of Erlangen):
Findings consistent with a glomus jugulare tumor right side (Type C1 according to Fisch and Valavanis).

19.02.2018:
Partial removal of glomus jugulare tumor right side (ENT Clinic, University of Erlangen).
Histology (ID No.: ****, Pathological Institute, University of Erlangen):
Paraganglioma of the glomus tympanicum (also known as a glomus tumor). No loss of SDHB.

21.02.2018:
Tumor board recommendation for head/neck tumors, University of Erlangen:
Radiation of the residual tumor.
Planned: Fractionated stereotactic radiotherapy in the area of the residual tumor at the right glomus jugulare.

Recipients:
Prof. Dr. med. Dr. h.c. Heinrich Iro, Director of the ENT Clinic, Waldstraße 1, 91054 Erlangen
Dr. med. Martin Weichmann, ENT Doctor, Marktplatz 8, 91781 Weißenburg

German

KI-Brief – Fall 3:

Sehr geehrter Herr Professor Iro,

wir berichten Ihnen über unsere gemeinsame Patientin, Frau ---------- , die sich am 27.02.2018 zu einem Aufklärungsgespräch in unserer strahlentherapeutischen Ambulanz vorstellte.

An Ihrer Klinik erfolgte am 19.02.2018 eine Teilresektion bei Glomus jugulare Tumor rechts. Im gemeinsamen Tumorboard für Kopf-/Hals-Tumore wurde die Empfehlung ausgesprochen, eine Strahlentherapie des Resttumors durchzuführen.
Wir haben die Patientin ausführlich über die geplante Strahlentherapie aufgeklärt und beraten und sie willigte in die Durchführung der Strahlentherapie ein.

Zum Abschluss des Gespräches haben wir folgende Termine koordiniert:

MRT-Schädel: 22.03.2018, 14.00 Uhr
Maskenanfertigung: 26.03.2018, 11.00 Uhr
Erste Bestrahlung am Novalis Classic: 03.04.2018, 14.00 Uhr

Im weiteren Verlauf erhalten Sie selbstverständlich erneut einen ausführlichen Bericht.

Wir bedanken uns für die kollegiale Zusammenarbeit und verbleiben

mit freundlichen kollegialen Grüßen

Unterschriften Prof. Dr. R. Fietkau — Dr. G. Lahmer
Direktor der Klinik — Oberarzt der Klinik

#	**Mache ein Kreuzchen für jede Kategorie!**	✗

Korrektheit: Bitte nicht die Datumsangaben bewerten, die nicht in den Inputdaten enthalten sind, diese sind eine Schätzung der KI aufgrund der Datumsangaben und des Zeitpunkts, an dem der Brief abgeschlossen wurde.

1	Schwerwiegende Fehler, Risiko für klin. Fehlentscheidungen	
2	Relevante Fehler, ohne klinische Auswirkung	
3	Kleinere Ungenauigkeiten, irrelevant für Patientenfall	
4	Der Brief enthält keine Fehler	

Notwendigkeit für Anpassungen:

1	Der Brief ist so unvollständig, dass es schneller ist, den Brief neu zu schreiben	
2	Der Brief benötigt größere Anpassungen > 1 min	
3	Der Brief benötigt kleiner Anpassungen <= 1 min	
4	Der Brief ist vollständig und benötigt keine Anpassungen	

Klinik- / Institutsspezifische Inhalte und Stil – Brief:

1	Keinerlei klinikspezifische Inhalte oder Anpassung an den lokalen Briefstil	

2	In sehr geringem Maße klinikspezifische Inhalte oder Anpassung an den lokalen Stil	
3	Der Brief enthält erhebliche klinischspezifische Inhalte oder Anpassungen an den lokalen Stil	
4	Der Brief bildet vollständig den Stil eines lokalen Briefes ab	

Einschätzung Praktischer Nutzen – Brief:

1	Kein Nutzen für die Brieferstellung	
2	Kleiner Nutzen für die Brieferstellung	
3	Mäßiger Nutzen für die Brieferstellung	
4	Großer Nutzen für die Brieferstellung	

English

AI Letter – Case 3:

Dear Professor Iro,

We are reporting on our mutual patient, Ms. **** ****, who presented herself on 27.02.2018 for an informational consultation in our radiation therapy outpatient clinic.

A partial resection of a right-sided Glomus jugulare tumor was performed at your clinic on 19.02.2018. The joint tumor board for head/neck tumors recommended radiation therapy of the residual tumor. We have thoroughly informed and advised the patient about the planned radiation therapy, and she consented to proceed with it.

At the end of the consultation, we coordinated the following appointments:

MRI of the skull: 22.03.2018, 14:00
Mask fabrication: 26.03.2018, 11:00
First radiation session on the Novalis Classic: 03.04.2018, 14:00

You will, of course, receive a detailed report on the further progress.

We thank you for the collegial cooperation and remain

with kind collegial regards,

Prof. Dr. R. Fietkau
Director of the clinic

Dr. G. Lahmer
Senior Physician of the Clinic

#	Make a mark for each category!	❌

Correctness: Please do not rate the date details that are not included in the input data; these are an estimate by the AI based on the dates and the time when the letter was completed.

1	Severe errors, risk for clinical misdecisions	
2	Relevant errors, no clinical impact	
3	Minor inaccuracies, irrelevant to the patient case	

4	The letter contains no errors	

Need for Adjustments:

1	The letter is so incomplete that it is faster to rewrite the letter	
2	The letter requires major adjustments > 1 min	
3	The letter requires minor adjustments <= 1 min	
4	The letter is complete and requires no adjustments	

Clinic-/Institute-Specific Content and Style – Letter:

1	No clinic-specific content or adjustment to local letter style	
2	Very little clinic-specific content or adjustment to local style	
3	The letter contains significant clinic-specific content or adjustments to the local style	
4	The letter fully reflects the style of a local letter	

Assessment Practical Utility – Letter:

1	No benefit for letter creation	
2	Small benefit for letter creation	
3	Moderate benefit for letter creation	
4	Great benefit for letter creation	

German

Original-Brief (Arzt) – Fall 3:

Sehr geehrter Herr Professor Iro,

Wir berichten Ihnen über unsere gemeinsame Patientin, Frau **** ****, die sich am 28.02.2018 zu einem Aufklärungsgespräch in unserer Strahlentherapeutischen Ambulanz vorstellte.

An Ihrer Klinik erfolgte am 19.02.2018 eine Teilresektion eines Glomus jugulare Tumors rechts.
Im gemeinsamen Tumorboard für Kopf-/Hals-Tumore wurde die Empfehlung ausgesprochen, eine Strahlentherapie durchzuführen.

Wir haben die Patientin ausführlich über die geplante Strahlentherapie aufgeklärt und beraten und sie willigte in die Durchführung der Strahlentherapie ein.
Zum Abschluss des Gespräches haben wir folgende Termine koordiniert:

1. Maskenanfertigung und Planungs-CT: 21.03.2018, 11.00 Uhr
2. MRT-Schädel (Kopfklinik): 23.03.2018, 16.30 Uhr
3. Erste Bestrahlung am Novalis Classic: 27.03.2018, 14.00 Uhr

Im weiteren Verlauf erhalten Sie selbstverständlich erneut einen ausführlichen Bericht.

Wir bedanken uns für die kollegiale Zusammenarbeit und verbleiben,

mit freundlichen kollegialen Grüßen

Prof. Dr. R. Fietkau
Direktor der Klinik

Dr. G. Lahmer
Oberarzt der Klinik

#	**Mache ein Kreuzchen für jede Kategorie!**	✘

Vergleich KI-Brief mit dem Originalbrief:

1	Der Originalbrief ist besser	
2	Originalbrief und KI-Brief sind gleich gut	
3	Der KI-Brief ist besser	

English

Original Letter (Doctor) – Case 3:

Dear Professor Iro,

We are reporting on our mutual patient, Ms. **** ****, who presented herself on 28.02.2018 for an informational consultation in our Radiation Therapy Outpatient Clinic.

A partial resection of a right-sided Glomus jugulare tumor was performed at your clinic on 19.02.2018. The joint tumor board for head and neck tumors recommended proceeding with radiation therapy.

We have thoroughly informed and advised the patient about the planned radiation therapy, and she consented to undergo the treatment. At the conclusion of our discussion, we coordinated the following appointments:

1- Mask fabrication and planning CT: 21.03.2018, 11:00 AM
2- MRI of the skull (Head Clinic): 23.03.2018, 4:30 PM
3- First radiation session on the Novalis Classic: 27.03.2018, 2:00 PM

You will, of course, receive a detailed report on the further progress.

We thank you for the collegial cooperation and remain,

with kind collegial regards,

Prof. Dr. R. Fietkau
Director of the clinic

Dr. G. Lahmer
Senior Physician of the Clinic

#	**Make a mark for each category!**	✘

Comparison of AI letter with the original letter:

1	The original letter is better	
2	The original letter and the AI letter are equally good	
3	The AI letter is better	

German

Modell-Input 4:

Dokument Abgeschlossen am: 22.12.2022
Abgezeichnet von: Sabrina Pirner

Briefkopf ST-HSA
Ansprechpartner Dr. med. S. Schnellhardt

Betreff:
Herrn **** ****, geb. --.--.----
910** ***

Diagnose:
V.a. metachron ossär metastasiertes Urothelkarzinom der Harnblase mind. pT2 cN0 cM1 L0 V0 G3 ED 07/22

Nebendiagnosen:
Z.n. Steinabgang rechts 2003
Glaukom
AHT

Tumorspezifische Anamnese:
07/22 Urologie Waldkrankenhaus
ED Urothelkarzinom nach TUR-B bei Makrohämaturie
Histologie: Wachstum und Infiltration in Muscularis propria, G3, mindestens pT2 L0 V0, TPS 0.5%, CPS 2
28.07.2022:
Thorax- /Abdomen-CT Nach TUR-Blase bei V.a. Urothelkarzinom ist zwischen postoperativen Veränderungen und Resttumor nicht sicher zu differenzieren.
V.a. Hämangiom im Lebersegment IVa/VIII, sonographische Korrelation empfohlen.
Keine suspekten Lymphknoten.
08/22:
Empfehlung neoadj. Chemotherapie vor radikaler Zystektomie. Ablehnung durch Patient
08/22 - 11/22: MVZ Herzogenaurach, Dr. Henschel
6x BCG Induktion
12/22:
MRT Becken (extern): V.a. ossäre Metastasierung Os sacrum

geplant:

- Radiotherapie Os sacrum
- Tumorboard Urologie
- Systemtherapie
- Restaging CT + Skelettszintigrafie

Empfänger:
Dr. Gerhard Baumüller, Arzt für Allgemeinmedizin, Bamberger Str. 10, 96132 Schlüsselfeld
N: Prof. Dr. med. Bernd Wullich, Direktor der Urologischen Klinik, Krankenhausstraße 1, 91054 Erlangen

English

Model input 4:

Document Completed on: 22.12.2022
Signed off by: Sabrina Pirner

Letterhead ST-HAS
Contact Dr. med. S. Schnellhardt

Subject:
Mr. **** **, born **.**.****
910** ***

Diagnosis:
Suspected metachronous osseous metastasized urothelial carcinoma of the bladder, at least pT2 cN0 cM1 L0 V0 G3, initial diagnosis 07/22

Secondary Diagnoses:
History of kidney stone passage right side 2003
Glaucoma
Hypertension (AHT)

Tumor-specific history:

07/22 Urology Waldkrankenhaus
Initial diagnosis of urothelial carcinoma following TUR-B for macrohematuria
Histology: Growth and infiltration into the muscularis propria, G3, at least pT2 L0 V0, TPS 0.5%, CPS 2

28.07.2022:
Thorax/Abdomen CT post TUR-bladder with suspected urothelial carcinoma, unable to differentiate between postoperative changes and residual tumor.
Suspected hemangioma in liver segment IVa/VIII, ultrasound correlation recommended.
No suspicious lymph nodes.

08/22:
Recommendation for neoadjuvant chemotherapy prior to radical cystectomy. Declined by patient.
08/22 - 11/22: MVZ Herzogenaurach, Dr. Henschel
6x BCG induction

12/22:
MRI pelvis (external): Suspected osseous metastasis to the sacrum

Planned:

- Radiotherapy to the sacrum
- Urology tumor board
- Systemic therapy
- Restaging CT + bone scintigraphy

Recipients:

Dr. Gerhard Baumüller, General Practitioner, Bamberger Str. 10, 96132 Schlüsselfeld
N: Prof. Dr. med. Bernd Wullich, Director of the Urological Clinic, Krankenhausstraße 1, 91054 Erlangen

German

KI-Brief – Fall 4:

Sehr geehrte Frau Kollegin, sehr geehrter Herr Kollege,

vielen Dank für die Vorstellung des o.g. Patienten, der sich am 21.12.2022 in unserer Strahlentherapeutischen Ambulanz zum Erstgespräch vorstellte.

Bei Herrn **** besteht der Verdacht auf eine solitäre ossäre Metastase im Bereich des Os sacrums. Wir sehen daher die Indikation zur Radiotherapie ebendort. Im Verlauf ist eine Wiedervorstellung im hiesigen Tumorboard der Urologie zur Planung der weiteren systemischen Therapie geplant.

Wir erklärten Herrn **** die Indikation zur Durchführung der Therapie, mögliche auftretende Risiken sowie Nebenwirkungen und Therapiealternativen. Am Ende des Gesprächs willigte der Patient in die Durchführung des vorgeschlagenen Procederes ein sodass wir erste Termine zur Bestrahlungsplanung vereinbarten.

Nach Abschluss unserer Behandlung werden wir erneut ausführlich Bericht erstatten.

Mit freundlichen kollegialen Grüßen

Prof. Dr. R. Fietkau
Direktor der Klinik

Dr. G. Lahmer
Oberarzt der Klinik

Dr. S. Schnellhardt
Assistenzarzt der Klinik

#	Mache ein Kreuzchen für jede Kategorie!	✘

Korrektheit: Bitte nicht die Datumsangaben bewerten, die nicht in den Inputdaten enthalten sind, diese sind eine Schätzung der KI aufgrund der Datumsangaben und des Zeitpunkts, an dem der Brief abgeschlossen wurde.

1	Schwerwiegende Fehler, Risiko für klin. Fehlentscheidungen	
2	Relevante Fehler, ohne klinische Auswirkung	

3	Kleinere Ungenauigkeiten, irrelevant für Patientenfall	
4	Der Brief enthält keine Fehler	

Notwendigkeit für Anpassungen:

1	Der Brief ist so unvollständig, dass es schneller ist, den Brief neu zu schreiben	
2	Der Brief benötigt größere Anpassungen > 1 min	
3	Der Brief benötigt kleiner Anpassungen <= 1 min	
4	Der Brief ist vollständig und benötigt keine Anpassungen	

Klinik- / Institutsspezifische Inhalte und Stil – Brief:

1	Keinerlei klinikspezifische Inhalte oder Anpassung an den lokalen Briefstil	
2	In sehr geringem Maße klinikspezifische Inhalte oder Anpassung an den lokalen Stil	
3	Der Brief enthält erhebliche klinischspezifische Inhalte oder Anpassungen an den lokalen Stil	
4	Der Brief bildet vollständig den Stil eines lokalen Briefes ab	

Einschätzung Praktischer Nutzen – Brief:

1	Kein Nutzen für die Brieferstellung	
2	Kleiner Nutzen für die Brieferstellung	
3	Mäßiger Nutzen für die Brieferstellung	
4	Großer Nutzen für die Brieferstellung	

English

AI Letter – Case 4:

Dear Colleague,

Thank you for referring the aforementioned patient, who presented at our Radiation Therapy Outpatient Clinic for an initial consultation on 21.12.2022.

Mr. **** is suspected of having a solitary osseous metastasis in the sacrum area. Therefore, we see an indication for radiotherapy in that location. Subsequently, a re-presentation at our local Urology Tumor Board is planned to discuss further systemic therapy.

We explained to Mr. **** the indication for the therapy, potential risks, side effects, and alternative treatments. At the end of the conversation, the patient consented to proceed with the suggested treatment, and we arranged initial appointments for radiation planning.

Upon completion of our treatment, we will report back in detail.

With kind collegial regards,

Prof. Dr. R. Fietkau<br>Director of the clinic	Dr. G. Lahmer<br>Senior Physician of the Clinic	Dr. S. Schnellhardt<br>Assistant Physician at the Clinic

#	Make a mark for each category!	✘

Correctness: Please do not rate the date details that are not included in the input data; these are an estimate by the AI based on the dates and the time when the letter was completed.

1	Severe errors, risk for clinical misdecisions	
2	Relevant errors, no clinical impact	
3	Minor inaccuracies, irrelevant to the patient case	
4	The letter contains no errors	

Need for Adjustments:

1	The letter is so incomplete that it is faster to rewrite the letter	
2	The letter requires major adjustments > 1 min	
3	The letter requires minor adjustments <= 1 min	
4	The letter is complete and requires no adjustments	

Clinic-/Institute-Specific Content and Style – Letter:

1	No clinic-specific content or adjustment to local letter style	
2	Very little clinic-specific content or adjustment to local style	
3	The letter contains significant clinic-specific content or adjustments to the local style	
4	The letter fully reflects the style of a local letter	

Assessment Practical Utility – Letter:

1	No benefit for letter creation	
2	Small benefit for letter creation	
3	Moderate benefit for letter creation	
4	Great benefit for letter creation	

German

Original-Brief (Arzt) – Fall 4:

Sehr geehrte Frau Kollegin, sehr geehrter Herr Kollege,

Vielen Dank für die Vorstellung des o.g. Patienten, der sich am 14.12.2022 in unserer Strahlentherapeutischen Ambulanz zum Erstgespräch vorstellte.

Bei dem Patienten wurde extern der V.a. eine schmerzhafte ossäre Metastasierung des bekannten Urothelkarzinoms gestellt, daher sahen wir die Indikation zur Strahlentherapie.

Der Patient wurde ausführlich über den Ablauf der geplanten Behandlung sowie mögliche Risiken und Nebenwirkungen informiert, er erklärte sich mit dem geplanten Vorgehen einverstanden.

Wir haben folgende Termine vereinbart:

- Planungs CT 15.12.2022 9:00 Uhr
- Tagesklinik 16.12.2022 13:00 Uhr
- Ein Restaging CT wurde durch die Kollegen der Urologie am 19.12.2022 vereinbart.
- Skeletszintigrafie 23.12.2022 9:15 Uhr.

Diese Termine wurden dem Patienten bereits mitgeteilt.

Nach Abschluss unserer Behandlung werden wir erneut ausführlich Bericht erstatten.

Mit freundlichen kollegialen Grüßen

Prof. Dr. R. Fietkau<br>Direktor der Klinik	Dr. G. Lahmer<br>Oberarzt der Klinik	Dr. S. Schnellhardt<br>Assistenzarzt der Klinik

#	**Mache ein Kreuzchen für jede Kategorie!**	✘

Vergleich KI-Brief mit dem Originalbrief:

1	Der Originalbrief ist besser	
2	Originalbrief und KI-Brief sind gleich gut	
3	Der KI-Brief ist besser	

English

Original Letter (Doctor) – Case 4:

Thank you for referring the aforementioned patient, who presented at our Radiation Therapy Outpatient Clinic for an initial consultation on 14.12.2022.

The patient was externally suspected of having painful osseous metastasis from the known urothelial carcinoma, hence we saw the indication for radiation therapy.

The patient was thoroughly informed about the process of the planned treatment as well as potential risks and side effects, and he agreed to the proposed approach.

We have scheduled the following appointments:

- Planning CT on 15.12.2022 at 9:00 AM
- Day clinic on 16.12.2022 at 1:00 PM
- A restaging CT was scheduled by the colleagues from Urology for 19.12.2022.
- Bone scintigraphy on 23.12.2022 at 9:15 AM.

These appointments have already been communicated to the patient.

After the completion of our treatment, we will provide a detailed report again.

With kind collegial regards,

Prof. Dr. R. Fietkau<br>Director of the clinic	Dr. G. Lahmer<br>Senior Physician of the Clinic	Dr. S. Schnellhardt<br>Assistant Physician at the Clinic

#	**Make a mark for each category!**	✘

Comparison of AI letter with the original letter:

1	The original letter is better	
2	The original letter and the AI letter are equally good	

3	The AI letter is better	

German

Modell-Input 5:

Dokument Abgeschlossen am: 27.09.2021
Abgeschlossen von: Dr. med. Godehard Lahmer

Briefkopf ST-HSA
Ansprechpartner Shwetha Samala

Betreff
Herrn **** ****, geb. --.--.-----
954** ***

Diagnosen
Anaplastisches Schilddrüsenkarzinom cT4b cN0 cM1 (pulmonal)

Nebendiagnosen:
Arterielle Hypertonie

Tumorspezifische Anamnese
26.07.2021:
Thorax-CT nativ (Radiologisches Institut, Universität Erlangen):
Bekannte thyreoidale Raumforderung rechts mit Trachealdeviation nach links, z.B. Schilddrüsenkarzinom. Multiple Rundherde in allen Lungenlappen, V.a. pulmonale Filiae. Leberläsion im linken Lappen, z.B. Zyste. NB: Nebennieren-Inzidentalom rechts.

26.07.2021:
Grobnadelbiopsie in LA:
MSS gering differenziertes Schilddrüsenkarzinom vereinbar mit anaplastischem Karzinom.

28.07.2021:
Empfehlung Tumorboard Kopf-Hals-Tumore (HNO-Klinik, Universität Erlangen):
(Tumordebulking, ggf. ND bds, Panendoskopie, Tracheoskopie ggf. Tracheotomie, ggf. PEG)
EG Strahlenklinik heute (Induktionschemotherapie).

30.07.2021:
Panendoskopie mit Versuch der PEG-Anlage.

02.08.2021:
PEG-Anlage durch die Kollegen der Medizinischen Klinik 1.

Geplant: Postoperative Radiochemotherapie

Empfänger
Prof. Dr. med. Dr. h. c. Heinrich Iro, Direktor der HNO-Klinik, Waldstraße 1, 91054 Erlangen
N: Dr. med. Bernward Wünsche, HNO-Arzt, Kanalstraße 17, 95444 Bayreuth

English

Model input 5:

Document Completed on: 27.09.2021
Completed by: Dr. med. Godehard Lahmer

Letterhead ST-HSA
Contact Dr. med. Shwetha Samala

Subject:
Mr. **** **, born **.**.****
954** ***

Diagnoses:
Anaplastic Thyroid Carcinoma cT4b cN0 cM1 (pulmonary)

Secondary Diagnoses:
Arterial Hypertension

Tumor-Specific history:

26.07.2021:
Thorax CT without contrast (Radiological Institute, University of Erlangen):
Known thyroid mass on the right with tracheal deviation to the left, e.g., thyroid carcinoma. Multiple nodules in all lung lobes, suspected pulmonary metastases. Liver lesion in the left lobe, e.g., cyst. NB: Right adrenal incidentaloma.

26.07.2021:
Core needle biopsy under local anesthesia:
MSS poorly differentiated thyroid carcinoma consistent with anaplastic carcinoma.

28.07.2021:
Recommendation from the Tumor Board for Head and Neck Tumors (ENT Clinic, University of Erlangen):
(Tumor debulking, possibly bilateral neck dissection, panendoscopy, tracheoscopy possibly tracheotomy, possibly PEG)
Today's recommendation from the Radiotherapy Clinic (induction chemotherapy).

30.07.2021:
Panendoscopy with an attempt to place a PEG tube.

02.08.2021:
PEG placement by colleagues from Medical Clinic 1.

Planned: Postoperative radiochemotherapy

Recipients:

Prof. Dr. med. Dr. h. c. Heinrich Iro, Director of the ENT Clinic, Waldstraße 1, 91054 Erlangen
N: Dr. med. Bernward Wünsche, ENT Doctor, Kanalstraße 17, 95444 Bayreuth

German

KI-Brief – Fall 5:

Sehr geehrter Herr Professor Iro, sehr geehrte Kollegen,

wir berichten über unseren gemeinsamen Patienten, Herrn **** ****, der sich am 02.08.2021 in unserer strahlentherapeutischen Ambulanz zum Erstgespräch vorstellte.

Im gemeinsamen Tumorboard für HNO-Tumore wurde die Empfehlung ausgesprochen eine postoperative Radiochemotherapie durchzuführen.

In einem ausführlichen Aufklärungsgespräch erläuterten wir dem Patienten im Beisein seiner Ehefrau die Indikation zur Durchführung der Therapie, den Ablauf, möglich auftretende Risiken und Nebenwirkungen sowie Therapiealternativen. Der Patient willigte daraufhin in die Therapie ein.

Zum Abschluss des Erstgespräches vereinbarten wir einen entsprechenden Termin zur Bestrahlungsplanung.

Nach Abschluss unserer Therapie erhalten Sie einen ausführlichen Bericht.

Wir bedanken uns für die Zuweisung des Patienten und verbleiben

mit freundlichen kollegialen Grüßen

Prof. Dr. R. Fietkau
Direktor der Klinik

Dr. G. Lahmer
Oberarzt der Klinik

S. Samala
Assistenzärztin

#	**Mache ein Kreuzchen für jede Kategorie!**	✘

Korrektheit: Bitte nicht die Datumsangaben bewerten, die nicht in den Inputdaten enthalten sind, diese sind eine Schätzung der KI aufgrund der Datumsangaben und des Zeitpunkts, an dem der Brief abgeschlossen wurde.

1	Schwerwiegende Fehler, Risiko für klin. Fehlentscheidungen	
2	Relevante Fehler, ohne klinische Auswirkung	
3	Kleinere Ungenauigkeiten, irrelevant für Patientenfall	
4	Der Brief enthält keine Fehler	

Notwendigkeit für Anpassungen:

1	Der Brief ist so unvollständig, dass es schneller ist, den Brief neu zu schreiben	
2	Der Brief benötigt größere Anpassungen > 1 min	
3	Der Brief benötigt kleiner Anpassungen <= 1 min	
4	Der Brief ist vollständig und benötigt keine Anpassungen	

Klinik- / Institutsspezifische Inhalte und Stil – Brief:

1	Keinerlei klinikspezifische Inhalte oder Anpassung an den lokalen Briefstil	
2	In sehr geringem Maße klinikspezifische Inhalte oder Anpassung an den lokalen Stil	

3	Der Brief enthält erhebliche klinischspezifische Inhalte oder Anpassungen an den lokalen Stil	
4	Der Brief bildet vollständig den Stil eines lokalen Briefes ab	

Einschätzung Praktischer Nutzen – Brief:

1	Kein Nutzen für die Brieferstellung	
2	Kleiner Nutzen für die Brieferstellung	
3	Mäßiger Nutzen für die Brieferstellung	
4	Großer Nutzen für die Brieferstellung	

English

AI Letter – Case 5:

Dear Professor Iro, dear colleagues,

We are reporting on our mutual patient, Mr. **** ****, who presented himself on 02.08.2021 at our radiation therapy outpatient clinic for an initial consultation.

The joint tumor board for ENT tumors recommended proceeding with postoperative radiochemotherapy.

In a detailed consultation, we explained to the patient in the presence of his wife the indication for the therapy, the procedure, potential risks and side effects, as well as alternative treatments. The patient then consented to the therapy.

At the conclusion of the initial consultation, we scheduled an appropriate appointment for radiation planning.

After the completion of our therapy, you will receive a detailed report.

We thank you for the referral of the patient and remain

with kind collegial regards,

Prof. Dr. R. Fietkau<br>Director of the clinic	Dr. G. Lahmer<br>Senior Physician of the Clinic	S. Samala<br>Assistant Physician

#	**Make a mark for each category!**	✘

Correctness: Please do not rate the date details that are not included in the input data; these are an estimate by the AI based on the dates and the time when the letter was completed.

1	Severe errors, risk for clinical misdecisions	
2	Relevant errors, no clinical impact	
3	Minor inaccuracies, irrelevant to the patient case	
4	The letter contains no errors	

Need for Adjustments:

1	The letter is so incomplete that it is faster to rewrite the letter	
2	The letter requires major adjustments > 1 min	

3	The letter requires minor adjustments <= 1 min	
4	The letter is complete and requires no adjustments	

Clinic-/Institute-Specific Content and Style – Letter:

1	No clinic-specific content or adjustment to local letter style	
2	Very little clinic-specific content or adjustment to local style	
3	The letter contains significant clinic-specific content or adjustments to the local style	
4	The letter fully reflects the style of a local letter	

Assessment Practical Utility – Letter:

1	No benefit for letter creation	
2	Small benefit for letter creation	
3	Moderate benefit for letter creation	
4	Great benefit for letter creation	

German

Original-Brief (Arzt) – Fall 5:

Sehr geehrter Herr Professor Iro,

wir berichten über unseren gemeinsamen Patienten, Herrn **** ****, der sich am 28.07.2021 in unserer strahlentherapeutischen Ambulanz zum Erstgespräch vorstellte.

Im gemeinsamen Tumorboard für Kopf-Hals-Tumore wurde die Empfehlung ausgesprochen eine Induktionschemotherapie durchzuführen. Nach der Panendoskopie und Tumordebulking am 03.08.2021 wurde eine postoperative Radiochemotherapie empfohlen.

In einem ausführlichen Aufklärungsgespräch erläuterten wir dem Patienten die Indikation zur Durchführung der Therapie, den Ablauf, möglich auftretende Risiken und Nebenwirkungen sowie Therapiealternativen. Der Patient willigte daraufhin in die Therapie ein.

Zum Abschluss des Erstgespräches vereinbarten wir einen entsprechenden Termin zur Bestrahlungsplanung.

Nach Abschluss unserer Therapie erhalten Sie einen ausführlichen Bericht.

Wir bedanken uns für die Zuweisung des Patienten und verbleiben

mit freundlichen kollegialen Grüßen

Prof. Dr. R. Fietkau<br>Direktor der Klinik	Dr. G. Lahmer<br>Oberarzt der Klinik	S. Samala<br>Assistenzärztin

#	**Mache ein Kreuzchen für jede Kategorie!**	✘

Vergleich KI-Brief mit dem Originalbrief:

1	Der Originalbrief ist besser	
2	Originalbrief und KI-Brief sind gleich gut	
3	Der KI-Brief ist besser	

English

Original Letter (Doctor) – Case 5:

Dear Professor Iro,

We are reporting on our mutual patient, Mr. **** ****, who presented himself on 28.07.2021 at our radiation therapy outpatient clinic for an initial consultation.

The joint tumor board for head and neck tumors recommended induction chemotherapy. Following panendoscopy and tumor debulking on 03.08.2021, postoperative radiochemotherapy was advised.

In a detailed consultation, we explained to the patient the indication for the therapy, the procedure, potential risks and side effects, as well as alternative treatments. The patient then consented to the therapy.

At the conclusion of the initial consultation, we scheduled an appropriate appointment for radiation planning.

After the completion of our therapy, you will receive a detailed report.

We thank you for the referral of the patient and remain

with kind collegial regards,

Prof. Dr. R. Fietkau<br>Director of the clinic	Dr. G. Lahmer<br>Senior Physician of the Clinic	S. Samala<br>Assistant Physician

#	**Make a mark for each category!**	✘

Comparison of AI letter with the original letter:

1	The original letter is better	
2	The original letter and the AI letter are equally good	
3	The AI letter is better	

German

Modell-Input 6:

Dokument Abgeschlossen am: 05.11.2020
Abgeschlossen von: Dr. med. Godehard Lahmer

Briefkopf:
ST-HSA
Ansprechpartner:
Dr. Thomas Weissmann

Betreff:
Herrn **** ****, geb. --.------ ,
912** ***

Diagnosen:
Prostatakarzinom
cT1c cN0 cM0
Gleason-Score 7a
iPSA 9,43 ng/ml

Nebendiagnose: Arterielle Hypertonie

Tumorspezifische Anamnese:
01/2019: 12fach Prostatastanzbiopsie.
09.05.2019 -
19.06.2019:
Radiotherapie Prostata und Samenblasen (Strahlentherapie, Klinikum Bayreuth):
Einzeldosis 1,8 Gy, Gesamtdosis 50,4 Gy.

Geplant: Lokale Aufsättigung mittels interstitieller Brachytherapie in primär kurativer Intention.

Empfänger:
Prof. Dr. med. Ludwig Keilholz, Chefarzt der Strahlenklinik, Klinikum Bayreuth, Preuschwitzer Straße 101, 95445 Bayreuth
Dr. med. Rudolf Koob, Facharzt für Innere Medizin, Nürnberger Straße 2 , 91257 Pegnitz

English

Model input 6:

Document Completed on: 05.11.2020
Completed by: Dr. med. Godehard Lahmer

Letterhead:
ST-HSA
Contact:
Dr. Thomas Weissmann

Subject:
Mr. **** **, born **.**.****,
912** ***

Diagnoses:
Prostate Cancer
cT1c cN0 cM0
Gleason Score 7a
iPSA 9.43 ng/ml

Secondary Diagnosis:
Arterial Hypertension

Tumor-Specific history:
01/2019: 12-core prostate biopsy.
09.05.2019 -
19.06.2019:
Radiotherapy of the prostate and seminal vesicles (Radiotherapy Department, Klinikum Bayreuth):
Single dose 1.8 Gy, total dose 50.4 Gy.
Planned: Local boost via interstitial brachytherapy with primarily curative intent.

Recipients:
Prof. Dr. med. Ludwig Keilholz, Chief Physician of the Radiotherapy Clinic, Klinikum Bayreuth, Preuschwitzer Straße 101, 95445 Bayreuth
Dr. med. Rudolf Koob, Specialist in Internal Medicine, Nürnberger Straße 2, 91257 Pegnitz

German

KI-Brief – Fall 6:

Sehr geehrter Herr Professor Keilholz,

wir berichten über unseren gemeinsamen Patienten, Herrn **** ****, der sich am 18.09.2019 im Beisein seiner Ehefrau zum Erstgespräch in unserer strahlentherapeutischen Ambulanz vorstellte.

Bei Herrn **** war im Rahmen der Abklärung bei erhöhtem PSA-Wert ein Prostatakarzinom diagnostiziert worden. Entsprechend der Histologie wurde hier eine kurative Radiotherapie im Klinikum Bayreuth bis zu einer Dosis von 50,4 Gy durchgeführt.

Entsprechend der Empfehlung des Radioonkologen wird hier jetzt in Erlangen die interstitielle Dosisaufsättigung der Prostata durchgeführt werden.
In einem ausführlichen Aufklärungsgespräch erläuterten wir dem Patienten die Indikation zur Durchführung der Therapie, mögliche auftretende Risiken und Nebenwirkungen sowie etwaige Therapiealternativen. Nach umfangreicher Überlegung entschied sich der Patient zur Durchführung der Therapie in unserem Hause.

Es wurden entsprechende Termine zur Vorbereitung am 29.10.2019 um 09.00 Uhr, zur stationären Aufnahme am 04.11.2019 um 07.30 Uhr und zur Operation am 05.11.2019 um 07.30 Uhr vereinbart.

Nach Abschluss unserer Behandlung werden wir erneut berichten.

Mit freundlichen kollegialen Grüßen

Prof. Dr. R. Fietkau	Dr. G. Lahmer	Dr. Th. Weissmann
Direktor der Klinik	Oberarzt der Klinik	Assistenzarzt

#	**Mache ein Kreuzchen für jede Kategorie!**	✘

Korrektheit: Bitte nicht die Datumsangaben bewerten, die nicht in den Inputdaten enthalten sind, diese sind eine Schätzung der KI aufgrund der Datumsangaben und des Zeitpunkts, an dem der Brief abgeschlossen wurde.

1	Schwerwiegende Fehler, Risiko für klin. Fehlentscheidungen	
2	Relevante Fehler, ohne klinische Auswirkung	
3	Kleinere Ungenauigkeiten, irrelevant für Patientenfall	
4	Der Brief enthält keine Fehler	

Notwendigkeit für Anpassungen:

1	Der Brief ist so unvollständig, dass es schneller ist, den Brief neu zu schreiben	
2	Der Brief benötigt größere Anpassungen > 1 min	
3	Der Brief benötigt kleiner Anpassungen <= 1 min	
4	Der Brief ist vollständig und benötigt keine Anpassungen	

Klinik- / Institutsspezifische Inhalte und Stil – Brief:

1	Keinerlei klinikspezifische Inhalte oder Anpassung an den lokalen Briefstil	

2	In sehr geringem Maße klinikspezifische Inhalte oder Anpassung an den lokalen Stil	
3	Der Brief enthält erhebliche klinischspezifische Inhalte oder Anpassungen an den lokalen Stil	
4	Der Brief bildet vollständig den Stil eines lokalen Briefes ab	

Einschätzung Praktischer Nutzen – Brief:

1	Kein Nutzen für die Brieferstellung	
2	Kleiner Nutzen für die Brieferstellung	
3	Mäßiger Nutzen für die Brieferstellung	
4	Großer Nutzen für die Brieferstellung	

English

<u>AI Letter – Case 6:</u>

Dear Professor Keilholz,

We are reporting on our mutual patient, Mr. **** ****, who presented himself on 18.09.2019 along with his wife for an initial consultation at our radiation therapy outpatient clinic.

Mr. **** was diagnosed with prostate cancer during an evaluation for an elevated PSA level. According to the histology, a curative radiotherapy up to a dose of 50.4 Gy was carried out at Klinikum Bayreuth.

Following the recommendation of the radio-oncologist, interstitial dose escalation of the prostate will now be carried out here in Erlangen.

In a detailed consultation, we explained to the patient the indications for the therapy, potential risks and side effects, as well as possible therapy alternatives. After thorough consideration, the patient decided to undergo the therapy at our facility.

Appointments have been scheduled for preparation on 29.10.2019 at 09:00 AM, for hospital admission on 04.11.2019 at 07:30 AM, and for the surgery on 05.11.2019 at 07:30 AM.

We will report again upon completion of our treatment.

With kind collegial regards,

Prof. Dr. R. Fietkau<br>Director of the clinic	Dr. G. Lahmer<br>Senior Physician of the Clinic	Dr. Th. Weissmann<br>Assistant Physician

#	**Make a mark for each category!**	✘

Correctness: Please do not rate the date details that are not included in the input data; these are an estimate by the AI based on the dates and the time when the letter was completed.

1	Severe errors, risk for clinical misdecisions	
2	Relevant errors, no clinical impact	
3	Minor inaccuracies, irrelevant to the patient case	
4	The letter contains no errors	

Need for Adjustments:

1	The letter is so incomplete that it is faster to rewrite the letter	
2	The letter requires major adjustments > 1 min	
3	The letter requires minor adjustments <= 1 min	
4	The letter is complete and requires no adjustments	

Clinic-/Institute-Specific Content and Style – Letter:

1	No clinic-specific content or adjustment to local letter style	
2	Very little clinic-specific content or adjustment to local style	
3	The letter contains significant clinic-specific content or adjustments to the local style	
4	The letter fully reflects the style of a local letter	

Assessment Practical Utility – Letter:

1	No benefit for letter creation	
2	Small benefit for letter creation	
3	Moderate benefit for letter creation	
4	Great benefit for letter creation	

German

Original-Brief (Arzt) – Fall 6:

Sehr geehrter Herr Professor Keilholz,

wir berichten über unseren gemeinsamen Patienten, Herrn **** ****, der sich am 21.06.2019 in unserer strahlentherapeutischen Ambulanz zum Erstgespräch vorstellte.

Bei Herrn **** war ein Zufallsbefund eines Prostatakarzinoms nachgewiesen worden.
Auf Wunsch des Patienten erfolgte nun in Kooperation mit der Strahlentherapie in Bayreuth ein kuratives Konzept. Es war bereits eine Radiotherapie der Prostata und Samenblasen bis 50,4 Gy erfolgt.

Nun soll mittels Brachytherapie die Dosisaufsättigung in primär kurativer Intention erfolgen.
Der Patient wurde in einem ausführlichen Aufklärungsgespräch von Herrn Professor Strnad bezüglich der Indikation zur Durchführung der Therapie, den Ablauf, mögliche auftretende Risiken und Nebenwirkungen sowie Therapiealternativen aufgeklärt. Der Patient willigte daraufhin in die Therapie ein.

Zum Abschluss des Erstgespräches vereinbarten wir entsprechende Termine zur Bestrahlungsplanung:
24.06.2019: Vorbereitungstermin (Blutentnahme, EKG, Anästhesieaufklärung)
26.06.2019: OP-Termin mit 3tägigem Aufenthalt

Nach Abschluss unserer Behandlung erhalten Sie einen ausführlichen Bericht.

Wir bedanken uns für die Zuweisung des Patienten und verbleiben

mit freundlichen kollegialen Grüßen

Prof. Dr. R. Fietkau	Dr. G. Lahmer	Dr. Th. Weissmann
Direktor der Klinik	Oberarzt der Klinik	Assistenzarzt

#	**Mache ein Kreuzchen für jede Kategorie!**	✘

Vergleich KI-Brief mit dem Originalbrief:

1	Der Originalbrief ist besser	
2	Originalbrief und KI-Brief sind gleich gut	
3	Der KI-Brief ist besser	

English

Original Letter (Doctor) – Case 6:

Dear Professor Keilholz,

We are reporting on our mutual patient, Mr. **** ****, who presented himself on 21.06.2019 at our radiation therapy outpatient clinic for an initial consultation.

Mr. **** had an incidental finding of prostate cancer. At the patient's request, a curative approach has now been implemented in cooperation with the radiotherapy department in Bayreuth. A radiotherapy of the prostate and seminal vesicles up to 50.4 Gy has already been carried out.

Now, dose escalation via brachytherapy is planned with a primarily curative intent.

The patient was thoroughly informed in a detailed consultation by Professor Strnad about the indications for the therapy, the process, potential risks and side effects, as well as alternative treatments. The patient then consented to the therapy.

At the conclusion of the initial consultation, we scheduled the following appointments for radiation planning:
24.06.2019: Preparation appointment (blood draw, EKG, anesthesia consultation)
26.06.2019: Surgery date with a 3-day stay

Upon completion of our treatment, you will receive a detailed report.

We thank you for the referral of the patient and remain

with kind collegial regards,

Prof. Dr. R. Fietkau
Director of the clinic

Dr. G. Lahmer
Senior Physician of the Clinic

Dr. Th. Weissmann
Assistant Physician

#	**Make a mark for each category!**	✘

Comparison of AI letter with the original letter:

1	The original letter is better	
2	The original letter and the AI letter are equally good	
3	The AI letter is better	

German

Modell-Input 7:

Dokument Abgeschlossen am: 28.11.2017
Abgeschlossen von: Dr. med. Godehard Lahmer

Briefkopf ST-HSA
Ansprechpartner: Dr. Godehard Lahmer

Betreff
Herrn **** ****, geb. **.**.****
907** Fürth

Diagnosen:
Hyphophysenadenomrezidiv STX, Prolaktin, TSH, FSH, LH neg.
ACTH mittel pos. (+++)

Nebendiagnosen:
Z. n. Karpaltunnel-OP rechts
Z. n. Schulter-OP rechts
Nikotinabusus

Tumorspezifische Anamnese:
16.04.2007: Transsphenoidale OP (Klinikum Nürnberg Süd).
Histologie:
Hypophysenadenom.
28.05.2015: Subtotale transsphenoidale Re-Operation (PD Dr. Kreutzer, Nürnberg).
Histologie:
ACTH-expressierendes Adenom.
17.11.2017: Transkranielle Re-Operation (Neurochirurgische Klinik, Universität Erlangen).
Histologie (Neuropathologisches Institut, Universität Erlangen):
Rest-/Rezidivanteile eines Hypophysenadenoms mit immunhistochemisch nachweisbarer Expression von ACTH und grenzwertig erhöhter Proliferations-aktivität (silent kortikotropes Adenom).
STX, Prolaktin, TSH, FSH, LH neg.
ACTH mittel pos. (+++)
Geplant: Fraktioniert stereotaktische Strahlentherapie.

Empfänger:
Prof. Dr. med. Michael Buchfelder, Direktor der Neurochirurgischen Klinik, Schwabachanlage 6, 91054 Erlangen
Dr. med. Werner Heininger, Facharzt für Allgemeinmedizin, Carlo-Schmid-Strasse 41 A, 90765 Fürth

English

Model input 7:

Document Completed on: 28.11.2017
Completed by: Dr. med. Godehard Lahmer

Letterhead:
ST-HSA
Contact:
Dr. Thomas Weissmann

Subject
Mr. **** **, born **.**.****
907** Fürth

Diagnoses:
Recurrent pituitary adenoma, STX, Prolactin, TSH, FSH, LH negative.
ACTH moderately positive (+++)

Secondary Diagnoses:
History of carpal tunnel surgery right
History of right shoulder surgery
Nicotine abuse

Tumor-Specific history:
16.04.2007: Transsphenoidal surgery (Klinikum Nürnberg Süd).
Histology:
Pituitary adenoma.
28.05.2015: Subtotal transsphenoidal re-operation (PD Dr. Kreutzer, Nürnberg).
Histology:
ACTH-expressing adenoma.
17.11.2017: Transcranial re-operation (Neurosurgical Clinic, University of Erlangen).
Histology (Neuropathological Institute, University of Erlangen):
Residual/recurrent parts of a pituitary adenoma with immunohistochemically detectable expression of ACTH and borderline elevated proliferative activity (silent corticotropic adenoma).
STX, Prolactin, TSH, FSH, LH negative.
ACTH moderately positive (+++)
Planned: Fractionated stereotactic radiotherapy.

Recipients:
Prof. Dr. med. Michael Buchfelder, Director of the Neurosurgical Clinic, Schwabachanlage 6, 91054 Erlangen
Dr. med. Werner Heininger, General Practitioner, Carlo-Schmid-Strasse 41 A, 90765 Fürth

German

KI-Brief – Fall 7:

Sehr geehrter Herr Professor Buchfelder,

wir berichten Ihnen über unseren gemeinsamen Patienten, Herrn **** ****, der sich am 27.11.2017 zu einem Aufklärungsgespräch in unserer strahlentherapeutischen Privatambulanz vorstellte.

Der Patient wurde bereits zweimal an einem Hypophysenadenom reseziert. Aktuell ist eine fraktioniert stereotaktische Strahlentherapie geplant. Wir haben den Patienten ausführlich über die geplante Strahlentherapie aufgeklärt und beraten und er willigte in die Durchführung der Strahlentherapie ein.

Zum Abschluss des Gespräches haben wir folgende Termine koordiniert:

- MRT-Schädel (Kopfklinik): 29.11.2017, 19.00 Uhr
- Maskenanfertigung: 01.12.2017, 10.30 Uhr
- Planungs-CT: 04.12.2017, 10.00 Uhr
- Erste Bestrahlung am Novalis Classic: 11.12.2017, 15.00 Uhr

Im weiteren Verlauf erhalten Sie selbstverständlich erneut einen ausführlichen Bericht.

Wir bedanken uns für die kollegiale Zusammenarbeit und verbleiben

mit freundlichen kollegialen Grüßen

Prof. Dr. R. Fietkau
Direktor der Klinik

Dr. G. Lahmer
Oberarzt der Klinik

#	Mache ein Kreuzchen für jede Kategorie!	✘

Korrektheit: Bitte nicht die Datumsangaben bewerten, die nicht in den Inputdaten enthalten sind, diese sind eine Schätzung der KI aufgrund der Datumsangaben und des Zeitpunkts, an dem der Brief abgeschlossen wurde.

diese sind eine Schätzung der KI aufgrund der Datumsangaben und des Zeitpunkts, an dem der Brief abgeschlossen wurde.

1	Schwerwiegende Fehler, Risiko für klin. Fehlentscheidungen	
2	Relevante Fehler, ohne klinische Auswirkung	
3	Kleinere Ungenauigkeiten, irrelevant für Patientenfall	
4	Der Brief enthält keine Fehler	

Notwendigkeit für Anpassungen:

1	Der Brief ist so unvollständig, dass es schneller ist, den Brief neu zu schreiben	
2	Der Brief benötigt größere Anpassungen > 1 min	
3	Der Brief benötigt kleiner Anpassungen <= 1 min	

4	Der Brief ist vollständig und benötigt keine Anpassungen	

Klinik- / Institutsspezifische Inhalte und Stil – Brief:

1	Keinerlei klinikspezifische Inhalte oder Anpassung an den lokalen Briefstil	
2	In sehr geringem Maße klinikspezifische Inhalte oder Anpassung an den lokalen Stil	
3	Der Brief enthält erhebliche klinischspezifische Inhalte oder Anpassungen an den lokalen Stil	
4	Der Brief bildet vollständig den Stil eines lokalen Briefes ab	

Einschätzung Praktischer Nutzen – Brief:

1	Kein Nutzen für die Brieferstellung	
2	Kleiner Nutzen für die Brieferstellung	
3	Mäßiger Nutzen für die Brieferstellung	
4	Großer Nutzen für die Brieferstellung	

English

AI Letter – Case 7:

Dear Professor Buchfelder,

We are reporting on our mutual patient, Mr. **** ****, who presented himself on 27.11.2017 for an informational consultation at our private radiation therapy outpatient clinic.

The patient has previously undergone two resections for a pituitary adenoma. Currently, a fractionated stereotactic radiotherapy is planned. We have thoroughly informed and advised the patient about the planned radiotherapy, and he consented to the treatment.

At the conclusion of the consultation, we coordinated the following appointments:

MRI of the skull (Head Clinic): 29.11.2017, 7:00 PM
Mask fabrication: 01.12.2017, 10:30 AM
Planning CT: 04.12.2017, 10:00 AM
First radiation session on the Novalis Classic: 11.12.2017, 3:00 PM
You will, of course, receive a detailed report on the further progress.

We thank you for the collegial cooperation and remain

With kind collegial regards,

Prof. Dr. R. Fietkau
Director of the clinic

Dr. G. Lahmer
Senior Physician of the Clinic

#	**Make a mark for each category!**	✘

Correctness: Please do not rate the date details that are not included in the input data; these are an estimate by the AI based on the dates and the time when the letter was completed.

1	Severe errors, risk for clinical misdecisions	
2	Relevant errors, no clinical impact	
3	Minor inaccuracies, irrelevant to the patient case	
4	The letter contains no errors	

Need for Adjustments:

1	The letter is so incomplete that it is faster to rewrite the letter	
2	The letter requires major adjustments > 1 min	
3	The letter requires minor adjustments <= 1 min	
4	The letter is complete and requires no adjustments	

Clinic-/Institute-Specific Content and Style – Letter:

1	No clinic-specific content or adjustment to local letter style	
2	Very little clinic-specific content or adjustment to local style	

3	The letter contains significant clinic-specific content or adjustments to the local style	
4	The letter fully reflects the style of a local letter	

Assessment Practical Utility – Letter:

1	No benefit for letter creation	
2	Small benefit for letter creation	
3	Moderate benefit for letter creation	
4	Great benefit for letter creation	

German

Original-Brief (Arzt) – Fall 7:

Sehr geehrter Herr Professor Buchfelder,

wir berichten Ihnen über unseren gemeinsamen Patienten, Herrn **** ****, der sich am 27.11.2017 zu einem Aufklärungsgespräch in unserer Strahlentherapeutischen Privatambulanz vorstellte.

Der Patient wurde erstmalig im Jahr 2007 an einem Hypophysenadenom operiert. Eine transsphenoidale Re-Operation erfolgte dann im Mai 2015.
Zuletzt wurde der Patient an Ihrer Klinik transkraniell operiert am 17.11.2017.

Im gemeinsamen Tumorboard für ZNS-Tumore wurde am 23.11.2017 die Empfehlung ausgesprochen, eine Strahlentherapie durchzuführen.
Wir haben den Patienten ausführlich über die geplante Strahlentherapie aufgeklärt und beraten und er willigte in die Durchführung der Strahlentherapie ein.

Zum Abschluss des Gespräches wurden folgende Termine koordiniert:

- MRT in der Kopfklinik: 18.12.2017, 17.00 Uhr
- Maskenanfertigung und Planungs-CT: 19.12.2017, 10.00 Uhr
- Erste Bestrahlung am Novalis Classic: 04.01.2018, 14.00 Uhr

Im weiteren Verlauf erhalten Sie selbstverständlich erneut einen ausführlichen Bericht.

Wir bedanken uns für die kollegiale Zusammenarbeit und verbleiben

mit freundlichen kollegialen Grüßen

Prof. Dr. R. Fietkau
Direktor der Klinik

Dr. G. Lahmer
Oberarzt der Klinik

#	**Mache ein Kreuzchen für jede Kategorie!**	✘

Vergleich KI-Brief mit dem Originalbrief:

1	Der Originalbrief ist besser	
2	Originalbrief und KI-Brief sind gleich gut	
3	Der KI-Brief ist besser	

English

Original Letter (Doctor) – Case 7:

Dear Professor Buchfelder,

We are reporting on our mutual patient, Mr. **** ****, who presented himself on 27.11.2017 for an informational consultation at our radiation therapy outpatient clinic.

The patient was first operated on for a pituitary adenoma in 2007. A transsphenoidal re-operation was then carried out in May 2015. Most recently, the patient was operated on transcranially at your clinic on 17.11.2017.

At the joint tumor board for CNS tumors on 23.11.2017, it was recommended that radiation therapy be administered. We have thoroughly informed and advised the patient about the planned radiotherapy, and he consented to the treatment.

At the conclusion of the consultation, the following appointments were coordinated:

- MRI at the Head Clinic: 18.12.2017, 5:00 PM
- Mask fabrication and planning CT: 19.12.2017, 10:00 AM
- First radiation session on the Novalis Classic: 04.01.2018, 2:00 PM

You will, of course, receive a detailed report on the further progress.

We thank you for the collegial cooperation and remain

with kind collegial regards,

Prof. Dr. R. Fietkau
Director of the clinic

Dr. G. Lahmer
Senior Physician of the Clinic

#	**Make a mark for each category!**	✘

Comparison of AI letter with the original letter:

1	The original letter is better	
2	The original letter and the AI letter are equally good	
3	The AI letter is better	

German

<u>Modell-Input 8:</u>

Dokument Abgeschlossen am: 15.09.2015
Abgeschlossen von: PD Dr. med. Sabine Semrau

Briefkopf ST-HSA
Ansprechpartner Thomas Gryc

Betreff:
Frau **** ****, geb. **.**.****
904** Nürnberg

Diagnosen:
Mammakarzinom beidseits (ED 2011) Histo (li+re): ER >90%, PR >80%, Her2neu 3+
Tumorstadium: rechts pT1c (m) pTis N0 (0/2) L1 V0 G3 R0
links pTc pTis N0 (0/1) L1 V0 G3 R0
aktuell: mult. ossäre u extraossäre Metastasen
Frakturgefahr BW 12
schmerzhaft Os Ileum rechts
große Osteolyse BW8

Tumorspezifische Anamnese:
01/2012: Mastektomie beidseits (Universitätsfrauenklinik Erlangen).
10/2013: Axilläre Lymphknotenmetastase rechts mit Axilladissektion rechts.
06/2015: Rekurrensparese links neu aufgetreten.
09.06.2015: CT Thorax/ Abdomen:

> Lymphknotenmetastasen supraklavikulär beidseits sowie mediastinal am linken Hilus, beidseits pulmonale Metastasierung, V.a. hepatische Metastasen, BWK 12-Metastase.

28.08.2015: Skelettszintigrafie:

> Diffuse osteolytische Metastasierung mit teils randständig vermehrter Knochenstoffwechselaktivität. Hinterkantenbeteiligung der Osteolyse BWK 12. Komplette Osteolyse der 8. Rippe links lateral. Mehrspeichernde Osteolyse im Os ilium rechts, ISG-nah. Mehrspeichernde Osteolyse im Femurkopf rechts.
> Nachbefund der Histologie eines axillären Lymphknoten links:
> ER mäßig starke Expression in 70% der Tumorzellen, PR: mäßig strake Expression in 20% der Tumorzellen, HER2-Onkogen: negativ. Damit bestätigt sich die Diagnose eines wenig differenzierten, invasiven Mammakarzinoms.
> Proliferationsrate (MIB1-Antigen): 40%.

08.09.2015: Beginn der palliativen Chemotherapie mit Epirubicin 30 mg/m² KOF und Paclitaxel 80 mg/m² KOF q21d (Universitätsfrauenklinik Erlangen).
geplant: Palliative Strahlentherapie der BWK 12-Metastase, evtuell im Anschluss Strahlentherapie der schmerzhaften Metastase der 8. Rippe sowie Os ilium rechts.

Empfänger:
Prof. Dr. med. Matthias W. Beckmann, Direktor der Frauenklinik, Universitätsklinikum Erlangen, Universitätsstr. 21-23, 91054 Erlangen
Dr. med. Otto Trautmann, Spitalgasse 6, 90403 Nürnberg

English

Model input 8:

Document Completed on: 15.09.2015
Completed by: PD Dr. med. Sabine Semrau

Letterhead ST-HSA
Contact Thomas Gryc

Subject:
Ms. **** **, born **.**.****
904 Nuremberg

Diagnoses:
Bilateral breast cancer (initial diagnosis 2011) Histology (left+right): ER >90%, PR >80%, Her2neu 3+
Tumor stage: right pT1c (m) pTis N0 (0/2) L1 V0 G3 R0
left pTc pTis N0 (0/1) L1 V0 G3 R0
current: multiple osseous and extraosseous metastases
Fracture risk at T12
Painful Os Ilium on the right
Large osteolysis at T8

Tumor-Specific history:
01/2012: Bilateral mastectomy (University Women's Clinic Erlangen).
10/2013: Right axillary lymph node metastasis with right axillary dissection.
06/2015: Newly developed left recurrent paralysis.
09.06.2015: CT Thorax/Abdomen:
Lymph node metastases supraclavicularly on both sides and mediastinal at the left hilum, bilateral pulmonary metastasis, suspected hepatic metastases, T12 vertebra metastasis.
28.08.2015: Bone scintigraphy:

Diffuse osteolytic metastasis with partly peripheral increased bone metabolism activity. Posterior edge involvement of the osteolysis at T12. Complete osteolysis of the 8th rib left lateral.
Hyperaccumulating osteolysis in the right Os ilium, close to the sacroiliac joint.
Hyperaccumulating osteolysis in the right femoral head.
Reevaluation of the histology of a left axillary lymph node:
ER moderately strong expression in 70% of tumor cells, PR: moderately strong expression in 20% of tumor cells, HER2-oncogene: negative. This confirms the diagnosis of a poorly differentiated, invasive breast carcinoma.
Proliferation rate (MIB1 antigen): 40%.
08.09.2015: Start of palliative chemotherapy with Epirubicin 30 mg/m² BSA and Paclitaxel 80 mg/m² BSA q21d (University Women's Clinic Erlangen).
planned: Palliative radiotherapy of the T12 metastasis, possibly followed by radiotherapy of the painful metastasis of the 8th rib and right Os ilium.

Recipients:

Prof. Dr. med. Matthias W. Beckmann, Director of the Women's Clinic, University Hospital Erlangen, Universitätsstr. 21-23, 91054 Erlangen
Dr. med. Otto Trautmann, Spitalgasse 6, 90403 Nuremberg

German

<u>KI-Brief – Fall 8:</u>

Sehr geehrter Herr Professor Beckmann

wir berichten über unsere gemeinsame Patientin, Frau **** ****, die sich am 14.09.2015 in unserer strahlentherapeutischen Ambulanz zum Erstgespräch vorstellte.

Bei der Patientin mit einem diffus metastasierten Mammakarzinom besteht die Indikation zur palliativen Chemotherapie sowie zur palliativen Strahlentherapie der BWK 12-Metastase.

Wir haben mit der Patientin ausführlich über die geplante Behandlung, deren Möglichkeiten und evttuelle Komplikationen gesprochen. Sie zeigte sich mit der Durchführung der Strahlentherapie einverstanden und wir konnten alle zur Einleitung der Behandlung notwendigen Termine vereinbaren.

Selbstverständlich werden wir erneut nach Abschluss der Therapie berichten.

Mit freundlichen kollegialen Grüßen

Prof. Dr. R. Fietkau	PD Dr. S. Semrau	T. Gryc
Direktor der Klinik	Oberärztin der Klinik	Assistenzarzt der Klinik

#	**Mache ein Kreuzchen für jede Kategorie!**	✗

Korrektheit: Bitte nicht die Datumsangaben bewerten, die nicht in den Inputdaten enthalten sind, diese sind eine Schätzung der KI aufgrund der Datumsangaben und des Zeitpunkts, an dem der Brief abgeschlossen wurde.

1	Schwerwiegende Fehler, Risiko für klin. Fehlentscheidungen	
2	Relevante Fehler, ohne klinische Auswirkung	
3	Kleinere Ungenauigkeiten, irrelevant für Patientenfall	
4	Der Brief enthält keine Fehler	

Notwendigkeit für Anpassungen:

1	Der Brief ist so unvollständig, dass es schneller ist, den Brief neu zu schreiben	
2	Der Brief benötigt größere Anpassungen > 1 min	
3	Der Brief benötigt kleiner Anpassungen <= 1 min	
4	Der Brief ist vollständig und benötigt keine Anpassungen	

Klinik- / Institutsspezifische Inhalte und Stil – Brief:

1	Keinerlei klinikspezifische Inhalte oder Anpassung an den lokalen Briefstil	
2	In sehr geringem Maße klinikspezifische Inhalte oder Anpassung an den lokalen Stil	
3	Der Brief enthält erhebliche klinischspezifische Inhalte<br>oder Anpassungen an den lokalen Stil	

4	Der Brief bildet vollständig den Stil eines lokalen Briefes ab	

Einschätzung Praktischer Nutzen – Brief:

1	Kein Nutzen für die Brieferstellung	
2	Kleiner Nutzen für die Brieferstellung	
3	Mäßiger Nutzen für die Brieferstellung	
4	Großer Nutzen für die Brieferstellung	

English

AI Letter – Case 8:

Dear Professor Beckmann,

We are reporting on our mutual patient, Ms. **** ****, who presented herself on 14.09.2015 at our radiation therapy outpatient clinic for an initial consultation.

The patient, with diffuse metastatic breast cancer, is indicated for palliative chemotherapy and palliative radiotherapy of the T12 metastasis.

We have thoroughly discussed the planned treatment, its possibilities, and potential complications with the patient. She agreed to proceed with the radiotherapy, and we were able to schedule all necessary appointments to initiate treatment.

Naturally, we will report again after the completion of the therapy.

With kind collegial regards,

Prof. Dr. R. Fietkau
Director of the clinic

PD Dr. S. Semrau
Senior Physician of the Clinic

T. Gryc
Assistant Physician

#	**Make a mark for each category!**	✗

Correctness: Please do not rate the date details that are not included in the input data; these are an estimate by the AI based on the dates and the time when the letter was completed.

1	Severe errors, risk for clinical misdecisions	
2	Relevant errors, no clinical impact	
3	Minor inaccuracies, irrelevant to the patient case	
4	The letter contains no errors	

Need for Adjustments:

1	The letter is so incomplete that it is faster to rewrite the letter	
2	The letter requires major adjustments > 1 min	
3	The letter requires minor adjustments <= 1 min	
4	The letter is complete and requires no adjustments	

Clinic-/Institute-Specific Content and Style – Letter:

1	No clinic-specific content or adjustment to local letter style	
2	Very little clinic-specific content or adjustment to local style	
3	The letter contains significant clinic-specific content or adjustments to the local style	
4	The letter fully reflects the style of a local letter	

Assessment Practical Utility – Letter:

1	No benefit for letter creation	
2	Small benefit for letter creation	
3	Moderate benefit for letter creation	
4	Great benefit for letter creation	

German

Original-Brief (Arzt) – Fall 8:

Sehr geehrter Herr Professor Beckmann,

wir berichten über unsere gemeinsame Patientin, Frau **** ****, die sich am 14.09.2015 zu einem Erstgespräch in unserer strahlentherapeutischen Ambulanz zum Erstgespräch vorstellte.

Bei der Patientin mit Zustand nach Mammakarzinom beidseits (ED 2011) besteht nun, aufgrund einer hochgradig frakturgefährdeten osteolytischen Metastase BWK 12 mit Hinterkantenbeteiligung, die Indikation zur palliativen Strahlentherapie.

Wir haben mit der Patientin das geplante Vorgehen, die möglichen Komplikationen sowie die Therapieziele besprochen, sie und ihr Bruder zeigten sich mit dem Vorgehen einverstanden und wir konnten alle zur Bestrahlungsplanung, ggf. auch im Rahmen der SPIN-MEt-Studie, erforderlichen Termine vereinbaren.

Nach Ende der Behandlung werden wir selbstverständlich erneut ausführlich berichten.

Mit freundlichen kollegialen Grüßen

Prof. Dr. R. Fietkau<br>Direktor der Klinik	PD Dr. S. Semrau<br>Oberärztin der Klinik	T. Gryc<br>Assistenzarzt der Klinik

#	Mache ein Kreuzchen für jede Kategorie!	✘

Vergleich KI-Brief mit dem Originalbrief:

1	Der Originalbrief ist besser	
2	Originalbrief und KI-Brief sind gleich gut	
3	Der KI-Brief ist besser	

German

Original Letter (Doctor) – Case 8:

Dear Professor Beckmann,

We are reporting on our mutual patient, Ms. **** ****, who presented herself for an initial consultation at our radiation therapy outpatient clinic on 14.09.2015.

The patient, who has a history of bilateral breast cancer (initial diagnosis in 2011), now has an indication for palliative radiotherapy due to a highly fracture-prone osteolytic metastasis at T12 with posterior edge involvement.

We discussed the planned approach, possible complications, and treatment goals with the patient. She and her brother were in agreement with the approach, and we were able to schedule all necessary appointments for radiation planning, possibly also as part of the SPIN-MEt study.

We will, of course, provide a detailed report again at the end of the treatment.

With kind collegial regards,

Prof. Dr. R. Fietkau	PD Dr. S. Semrau	T. Gryc
Director of the clinic	Senior Physician of the Clinic	Assistant Physician

#	Make a mark for each category!	✘

Comparison of AI letter with the original letter:

1	The original letter is better	
2	The original letter and the AI letter are equally good	
3	The AI letter is better	

German

Modell-Input 9:

Dokument Abgeschlossen am: 30.07.2020
Abgezeichnet von: Dr. med. Hanno Zoske

Briefkopf ST-PRI
Ansprechpartner:
Dr. Hanno Zoske

Betreff:
Frau **** ****, geb. **.**.****
925** ***

Diagnosen
Rektumkarzinom unteres Drittel cT3 cN+ cM0

Nebendiagnosen:
Blande Leberzysten

Tumorspezifische Anamnese
01/2020: Blut im Stuhl.
Koloskopische Diagnostik mit Abtragung multipler Polypen u. a. distales Rektum-Drittel bei exophytischem Wachstum
Histopathologisch Adenom-Anteile mit hochgradigem Karzinom-Verdacht.
Diagnostisches Staging-Komplettierung MRT/CT/Endosonographie:
V. a. tiefsitzendes Rektumkarzinom, cT3 cN+ cM0.

21.01.2020: Interdisziplinärer Tumorkonferenzkonsens (Uniklinik Regensburg):
Neoadjuvante Radiochemotherapie bei kurativer Operation und zusätzlicher Appendektomie (Polypenresiduum).

02-03/2020: Definitive Radiochemotherapie (Strahlentherapie Regensburg):
1,8 Gy bis 50,4 Gy im Bereich des Rektums und entsprechendem Lymphabflussgebiet.
Chemotherapie:
2 Kurse 5-Fu.

04/2020: Patientin lehnt operative Resektion ab.
Konzeptänderung von neoadjuvant auf definitiv, entsprechend lokale Dosis-Aufsättigung im Bereich des Primärtumors bis 60,4 Gy + zusätzlich 2 Kurse FOLFOX.

06/2020: Rektoskopisch Restumor cT2 cN0.
Geplant: Evaluation der Möglichkeit einer interstitiellen Brachytherapie.

Empfänger:
Dr. med. Michael Allgäuer, Abteilung Strahlentherapie , Krankenhaus Barmherzige Brüder , Prüfeninger Straße 86, 93049 Regensburg

English

Model input 9:

Document Completed on: 30.07.2020
Signed off by: Dr. med. Hanno Zoske

Letterhead ST-PRI
Contact:
Dr. Hanno Zoske

Subject:
Ms. **** **, born **.**.****
925*** ***

Diagnoses:
Rectal carcinoma lower third cT3 cN+ cM0

Secondary Diagnoses:
Bland liver cysts

Tumor-Specific history:
01/2020: Blood in the stool.
Colonoscopic diagnosis with removal of multiple polyps, including the distal third of the rectum with exophytic growth. Histopathological adenoma parts with high-grade suspected carcinoma. Diagnostic staging completion MRI/CT/endosonography: Suspected deep-seated rectal carcinoma, cT3 cN+ cM0.

01/21/2020: Interdisciplinary tumor conference consensus (University Hospital Regensburg): Neoadjuvant radiochemotherapy for curative surgery and additional appendectomy (polyp residue).

02-03/2020: Definitive radiochemotherapy (radiotherapy Regensburg): 1.8 Gy to 50.4 Gy in the area of the rectum and corresponding lymph drainage area. Chemotherapy: 2 courses of 5-Fu.

04/2020: Patient refuses surgical resection. Change of concept from neoadjuvant to definitive, corresponding local dose escalation in the area of the primary tumor up to 60.4 Gy + additional 2 courses of FOLFOX.

06/2020: Rectoscopic residual tumor cT2 cN0. Planned: Evaluation of the possibility of interstitial brachytherapy.
Recipient:
Dr. med. Michael Allgäuer, Department of Radiotherapy, Krankenhaus Barmherzige Brüder, Prüfeninger Straße 86, 93049 Regensburg

German

KI-Brief – Fall 9:

Sehr geehrter Herr Dr. Allgäuer,

wir berichten über unsere gemeinsame Patientin, Frau **** ****, die sich am 29.07.2020 in unserer strahlentherapeutischen Ambulanz zum Erstgespräch vorstellte.

Bei der Patientin wurde ein Rektumkarzinom unter neoadjuvanter Radiochemotherapie definitiv bestrahlt. Die Patientin lehnte die empfohlene operative Resektion ab, so dass die Radiochemotherapie als definitiv angesehen wurde und entsprechend lokal dosis-aufgesättigt wurde.

Aktuell stellt sich Frau **** bei uns mit einer persistierenden Hämatochezie vor. In unserer gynäkologischen Untersuchung zeigt sich ein großer Tumor im Bereich des unteren Rektums, der die Darmwand im Bereich des unteren Rektums infiltriert.

Wir haben Frau **** über die möglichen Risiken sowie den zu erwartenden Nutzen einer interstitiellen Brachytherapie aufgeklärt und sie willigte in die Therapie ein.

Folgende Termine wurden für die Patientin bereits vereinbart:

06.08.2020 OP-Vorbereitung.
10.08.2020 OP.

Wir danken für die freundliche Zuweisung der Patientin und verbleiben

mit freundlichen kollegialen Grüßen

Prof. Dr. R. Fietkau<br>Direktor der Klinik	Prof. Dr. V. Strnad<br>Ltd. Oberarzt der Klinik	Dr. H. Zoske<br>Assistenzarzt der Klinik

#	**Mache ein Kreuzchen für jede Kategorie!**	✗

Korrektheit: Bitte nicht die Datumsangaben bewerten, die nicht in den Inputdaten enthalten sind, diese sind eine Schätzung der KI aufgrund der Datumsangaben und des Zeitpunkts, an dem der Brief abgeschlossen wurde.

1	Schwerwiegende Fehler, Risiko für klin. Fehlentscheidungen	
2	Relevante Fehler, ohne klinische Auswirkung	
3	Kleinere Ungenauigkeiten, irrelevant für Patientenfall	
4	Der Brief enthält keine Fehler	

Notwendigkeit für Anpassungen:

1	Der Brief ist so unvollständig, dass es schneller ist, den Brief neu zu schreiben	
2	Der Brief benötigt größere Anpassungen > 1 min	
3	Der Brief benötigt kleiner Anpassungen <= 1 min	
4	Der Brief ist vollständig und benötigt keine Anpassungen	

Klinik- / Institutsspezifische Inhalte und Stil – Brief:

1	Keinerlei klinikspezifische Inhalte oder Anpassung an den lokalen Briefstil	
2	In sehr geringem Maße klinikspezifische Inhalte oder Anpassung an den lokalen Stil	
3	Der Brief enthält erhebliche klinischspezifische Inhalte<br>oder Anpassungen an den lokalen Stil	
4	Der Brief bildet vollständig den Stil eines lokalen Briefes ab	

Einschätzung Praktischer Nutzen – Brief:

1	Kein Nutzen für die Brieferstellung	
2	Kleiner Nutzen für die Brieferstellung	
3	Mäßiger Nutzen für die Brieferstellung	
4	Großer Nutzen für die Brieferstellung	

English

AI Letter – Case 9:

Dear Dr. Allgäuer,

We are reporting on our mutual patient, Ms. **** ****, who presented herself on 29.07.2020 at our radiation therapy outpatient clinic for an initial consultation.

The patient was definitively irradiated for a rectal carcinoma using neoadjuvant chemoradiotherapy. The patient refused the recommended surgical resection, so that chemoradiotherapy was considered definitive and the dose was increased locally accordingly.

Ms. **** is currently presenting to us with persistent hematochezia. Our gynecological examination shows a large tumor in the area of the lower rectum that infiltrates the intestinal wall in the area of the lower rectum.

We informed Ms. **** about the possible risks and the expected benefits of interstitial brachytherapy and she agreed to the therapy.

The following appointments have already been made for the patient:
06.08.2020 preparation for brachytherapy procedure.
10.08.2020 brachytherapy implantation.

with kind collegial regards,

Prof. Dr. R. Fietkau
Director of the clinic

Prof. Dr. V. Strnad
Ltd. Senior Physician of the Clinic

Dr. H. Zoske
Assistant Physician

#	Make a mark for each category!	✗

Correctness: Please do not rate the date details that are not included in the input data; these are an estimate by the AI based on the dates and the time when the letter was completed.

1	Severe errors, risk for clinical misdecisions	
2	Relevant errors, no clinical impact	
3	Minor inaccuracies, irrelevant to the patient case	
4	The letter contains no errors	

Need for Adjustments:

1	The letter is so incomplete that it is faster to rewrite the letter	
2	The letter requires major adjustments > 1 min	
3	The letter requires minor adjustments <= 1 min	
4	The letter is complete and requires no adjustments	

Clinic-/Institute-Specific Content and Style – Letter:

1	No clinic-specific content or adjustment to local letter style	
2	Very little clinic-specific content or adjustment to local style	
3	The letter contains significant clinic-specific content or adjustments to the local style	
4	The letter fully reflects the style of a local letter	

Assessment Practical Utility – Letter:

1	No benefit for letter creation	
2	Small benefit for letter creation	
3	Moderate benefit for letter creation	
4	Great benefit for letter creation	

German

Original-Brief (Arzt) – Fall 9:

Sehr geehrter Herr Kollege Allgäuer,

wir berichten über unsere gemeinsame Patientin, Frau **** ****, die sich am 10.07.2020 in unserer strahlentherapeutischen Ambulanz zum Erstgespräch vorstellte.

Bei der Patientin wurde im Klinikum Regensburg die neoadjuvante Radiochemotherapie des bei ihr diagnostizierten Rektumkarzinoms im o.g. Stadium durchgeführt.
Frau **** lehnt jedoch eine operative Resektion des Rektumkarzinoms aufgrund des Risiko einer möglichen Stoma-Anlage ab.
Die Patientin hat Angst, dass das verwendete Pflaster im Stoma-Bereich eine starke allergische Reaktion bei ihr auslösen würde.

Wir haben eine klinische, sowie sonographische Untersuchung der Patientin durchgeführt. Hier zeigte sich der bereits beschriebene Tumor von ca. 2 cm bis 3 cm Größe bei 5 Uhr SSL.

Eine Brachytherapie wäre grundsätzlich technisch durchführbar, jedoch besteht auch bei der Brachytherapie das Risiko, dass aufgrund von Toxizität, hervorgerufen durch die Brachytherapie, eine Stoma-Anlage erfolgen muss.

Aus diesem Grunde steht Frau **** eben auch dieser Therapieoption aktuell ablehnend gegenüber, so dass wir sie ans Klinikum Regensburg zurückverwiesen haben zur Reevaluation einer operativen Resektion des bei ihr bekannten Rektumkarzinoms.

Eine Wiedervorstellung bei Meinungsänderung der Patientin ist gerne jederzeit möglich.

Wir danken für die freundliche Zuweisung und

Verbleiben mit freundlichen kollegialen Grüßen,

Prof. Dr. R. Fietkau
Direktor der Klinik

Prof. Dr. V. Strnad
Ltd. Oberarzt der Klinik

Dr. H. Zoske
Assistenzarzt der Klinik

#	**Mache ein Kreuzchen für jede Kategorie!**	✘

Vergleich KI-Brief mit dem Originalbrief:

1	Der Originalbrief ist besser	
2	Originalbrief und KI-Brief sind gleich gut	
3	Der KI-Brief ist besser	

English

Original Letter (Doctor) – Case 9:

Dear Colleague Allgäuer,

We are reporting on our mutual patient, Ms. **** ****, who presented herself on 10.07.2020 at our radiation therapy outpatient clinic for an initial consultation.

At Klinikum Regensburg, the patient underwent neoadjuvant radiochemotherapy for the rectal carcinoma she was diagnosed with in the above-mentioned stage at the Regensburg Clinic.

However, Ms. ***** refuses surgical resection of the rectal carcinoma due to the risk of a possible colostomy. The patient is afraid that the plaster used in the colostomy area would trigger a strong allergic reaction.

We carried out a clinical and sonographic examination of the patient. This showed the previously described tumor, measuring approximately 2 cm to 3 cm, at 5 o'clock SSL.

Brachytherapy would in principle be technically feasible, but even with brachytherapy there is a risk that a colostomy will be necessary due to toxicity caused by the brachytherapy.

For this reason, Ms. **** is currently opposed to this treatment option, so we have referred her back to the Regensburg Clinic for re-evaluation of surgical resection of the known rectal carcinoma.

If the patient changes her mind, she can be re-presented at any time.

We thank you for the kind referral and

Remain with kind collegial regards,

Prof. Dr. R. Fietkau<br>Director of the clinic	Prof. Dr. V. Strnad<br>Ltd. Senior Physician of the Clinic	Dr. H. Zoske<br>Assistant Physician

#	**Make a mark for each category!**	✘

Comparison of AI letter with the original letter:

1	The original letter is better	
2	The original letter and the AI letter are equally good	
3	The AI letter is better	

German

Modell-Input 10:

Patientendaten:
Dokument Abgeschlossen am: 19.07.2018
Abgeschlossen von: Dr. med. Godehard Lahmer

Briefkopf ST-HSA
Ansprechpartner: Dr. Godehard Lahmer

Betreff:
Frau **** ****, geb. --.--.----
905** ***

Diagnosen:
Diagnose: Glioblastom WHO-Grad IV rechts frontal, IDH-1-Wildtyp
Nebendiagnosen:
Z.n. Schilddrüsen-OP
Hypothyreose
Anamnestisch Z.n. Röntgentherapie der Ferse (Fürth)
Anamnestisch Z.n. TUR-Blase mit Chemoinstillation (Fürth)
Z.n. Hysterektomie
Art. Hypertonus

Tumorspezifische Anamnese:
22.06.2018: Navigationsgestützte Anlage einer Rickhamkapsel rechts frontal (Neurochirurgische Klinik, Universität Erlangen).
29.06.2018: Rechts frontale Trepanation und Resektion des Prozesses unter Zuhilfenahme der intraoperativen Bildgebung (Neurochirurgische Klinik, Universität Erlangen).
Histologie (Neuropathologisches Institut, Universität Erlangen):
Glioblastom, IDH-1-Wildtyp, WHO-Grad IV.
05.07.2018: Tumorboardempfehlung ZNS-Tumore, Universität Erlangen:
Kombinierte Radiochemotherapie nach STUPP-Protokoll.

Geplant:
1. Postoperative Radiochemotherapie mit Temozolomid analog dem STUPP-Protokoll
2. Anschließend Erhaltungschemotherapie mit Temozolomid und Angebot für
Tumor treating fields.
3. Wenn möglich Teilnahme an der GlioCMV-Studie.

Empfänger:
Prof. Dr. med. Michael Buchfelder, Direktor der Neurochirurgischen Klinik, Schwabachanlage 6, 91054 Erlangen
N: Dr. med. Gernot Vogt-Ladner, Facharzt für Innere Medizin, Lehmusstraße 22 , 90766 Fürth

English

Model input 10:

Document Completed on: 19.07.2018
Completed by: Dr. med. Godehard Lahmer

Letterhead ST-HSA
Contact: Dr. Godehard Lahmer

Subject:
Ms. **** **, born --.--.----
905*** ***

Diagnoses:
Diagnosis: Right frontal glioblastoma, WHO Grade IV, IDH-1 wild-type
Secondary Diagnoses:
History of thyroid surgery
Hypothyroidism
Historical x-ray therapy of the heel (Fürth)
Historical TUR-Bladder with chemoinstillation (Fürth)
History of hysterectomy
Arterial hypertension

Tumor-Specific history:
22.06.2018: Navigation-assisted placement of a Rickham capsule right frontal (Neurosurgical Clinic, University of Erlangen).
29.06.2018: Right frontal trepanation and resection of the process using intraoperative imaging (Neurosurgical Clinic, University of Erlangen).
Histology (Neuropathological Institute, University of Erlangen):
Glioblastoma, IDH-1 wild-type, WHO Grade IV.
05.07.2018: CNS Tumor Board recommendation, University of Erlangen:
Combined radiochemotherapy according to the STUPP protocol.

Planned:
1- Postoperative radiochemotherapy with Temozolomide according to the STUPP protocol.
2- Subsequent maintenance chemotherapy with Temozolomide and offer for Tumor Treating Fields.
3- Possible participation in the GlioCMV study.

Recipients:
Prof. Dr. med. Michael Buchfelder, Director of the Neurosurgical Clinic, Schwabachanlage 6, 91054 Erlangen
N: Dr. med. Gernot Vogt-Ladner, Specialist in Internal Medicine, Lehmusstraße 22, 90766 Fürth

German

KI-Brief – Fall 10:

Sehr geehrter Herr Professor Buchfelder,

wir berichten Ihnen über unsere gemeinsame Patientin, Frau **** ****, die sich am 18.07.2018 zu einem Aufklärungsgespräch in unserer strahlentherapeutischen Ambulanz vorstellte.

An Ihrer Klinik erfolgte am 29.06.2018 eine rechts frontale Trepanation und Tumorresektion. Histopathologisch zeigte sich ein Glioblastoma multiforme, WHO-Grad IV. Nun besteht die Indikation zur postoperativen simultanen Radiochemotherapie mit Temozolomid.

Wir haben die Patientin ausführlich über die geplante Strahlentherapie aufgeklärt und beraten und sie willigte in die Durchführung der Strahlentherapie ein.

Zum Abschluss des Gespräches haben wir folgende Termine koordiniert:

- Tagesklinik: 30.07.2018, 10.30 Uhr
- Maskenanfertigung: 01.08.2018, 10.30 Uhr
- MRT-Schädel: 03.08.2018, 12.45 Uhr

Im weiteren Verlauf erhalten Sie selbstverständlich erneut einen ausführlichen Bericht.

Wir bedanken uns für die kollegiale Zusammenarbeit und verbleiben

mit freundlichen kollegialen Grüßen

Prof. Dr. R. Fietkau
Direktor der Klinik

Dr. G. Lahmer
Oberarzt der Klinik

#	**Mache ein Kreuzchen für jede Kategorie!**	✗

Korrektheit: Bitte nicht die Datumsangaben bewerten, die nicht in den Inputdaten enthalten sind, diese sind eine Schätzung der KI aufgrund der Datumsangaben und des Zeitpunkts, an dem der Brief abgeschlossen wurde.

1	Schwerwiegende Fehler, Risiko für klin. Fehlentscheidungen	
2	Relevante Fehler, ohne klinische Auswirkung	
3	Kleinere Ungenauigkeiten, irrelevant für Patientenfall	
4	Der Brief enthält keine Fehler	

Notwendigkeit für Anpassungen:

1	Der Brief ist so unvollständig, dass es schneller ist, den Brief neu zu schreiben	
2	Der Brief benötigt größere Anpassungen > 1 min	
3	Der Brief benötigt kleiner Anpassungen <= 1 min	
4	Der Brief ist vollständig und benötigt keine Anpassungen	

Klinik- / Institutsspezifische Inhalte und Stil – Brief:

1	Keinerlei klinikspezifische Inhalte oder Anpassung an den lokalen Briefstil	
2	In sehr geringem Maße klinikspezifische Inhalte oder Anpassung an den lokalen Stil	
3	Der Brief enthält erhebliche klinischspezifische Inhalte oder Anpassungen an den lokalen Stil	
4	Der Brief bildet vollständig den Stil eines lokalen Briefes ab	

Einschätzung Praktischer Nutzen – Brief:

1	Kein Nutzen für die Brieferstellung	
2	Kleiner Nutzen für die Brieferstellung	
3	Mäßiger Nutzen für die Brieferstellung	
4	Großer Nutzen für die Brieferstellung	

English

AI Letter – Case 10:

Dear Professor Buchfelder,

We are reporting on our mutual patient, Ms. **** ****, who presented herself on 18.07.2018 for an informational consultation at our radiation therapy outpatient clinic.

At your clinic, a right frontal trepanation and tumor resection were performed on 29.06.2018. Histopathologically, it was confirmed as glioblastoma multiforme, WHO Grade IV. There is now an indication for postoperative simultaneous radiochemotherapy with temozolomide.

We have thoroughly informed and advised the patient about the planned radiotherapy, and she consented to proceed with the treatment.

At the conclusion of the consultation, we coordinated the following appointments:

- Day Clinic: 30.07.2018, 10:30 AM
- Mask Fabrication: 01.08.2018, 10:30 AM
- MRI of the Skull: 03.08.2018, 12:45 PM

You will, of course, receive a detailed report on the further progress.

We thank you for the collegial cooperation and remain

with kind collegial regards,

Prof. Dr. R. Fietkau
Director of the clinic

Dr. G. Lahmer
Senior Physician of the Clinic

#	**Make a mark for each category!**	✘

Correctness: Please do not rate the date details that are not included in the input data; these are an estimate by the AI based on the dates and the time when the letter was completed.

1	Severe errors, risk for clinical misdecisions	
2	Relevant errors, no clinical impact	
3	Minor inaccuracies, irrelevant to the patient case	
4	The letter contains no errors	

Need for Adjustments:

1	The letter is so incomplete that it is faster to rewrite the letter	
2	The letter requires major adjustments > 1 min	
3	The letter requires minor adjustments <= 1 min	
4	The letter is complete and requires no adjustments	

Clinic-/Institute-Specific Content and Style – Letter:

1	No clinic-specific content or adjustment to local letter style	
2	Very little clinic-specific content or adjustment to local style	
3	The letter contains significant clinic-specific content or adjustments to the local style	
4	The letter fully reflects the style of a local letter	

Assessment Practical Utility – Letter:

1	No benefit for letter creation	
2	Small benefit for letter creation	
3	Moderate benefit for letter creation	
4	Great benefit for letter creation	

<u>Original-Brief (Arzt) – Fall 10:</u>

Sehr geehrter Herr Professor Buchfelder,
wir berichten Ihnen über unsere gemeinsame Patientin, Frau **** ****, die sich am 18.07.2018 zusammen mit ihren zwei Töchtern in unserer strahlentherapeutischen Ambulanz vorstellte.

An Ihrer Klinik erfolgte am 29.06.2018 eine Tumorresektion rechts frontal. Histopathologisch zeigte sich ein Glioblastoma multiforme, WHO-Grad IV.
Nun besteht die Indikation zur postoperativen simultanen Radiochemotherapie.

Wir haben die Patientin ausführlich über die geplante Strahlentherapie aufgeklärt und beraten und sie willigte in die Durchführung der Strahlentherapie ein.
Zum Abschluss des Gespräches haben wir folgende Termine koordiniert:

- Maskenanfertigung: 26.07.2018, 09:00 Uhr
- MRT-Schädel: 26.07.2018, 19:30 Uhr
- Tagesklinik: 30.07.2018, 11:00 Uhr

Im weiteren Verlauf erhalten Sie selbstverständlich erneut einen ausführlichen Bericht.
Wir bedanken uns für die kollegiale Zusammenarbeit und verbleiben

mit freundlichen kollegialen Grüßen

Prof. Dr. R. Fietkau
Direktor der Klinik

Dr. G. Lahmer
Oberarzt der Klinik

#	**Mache ein Kreuzchen für jede Kategorie!**	✘

Vergleich KI-Brief mit dem Originalbrief:

1	Der Originalbrief ist besser	
2	Originalbrief und KI-Brief sind gleich gut	
3	Der KI-Brief ist besser	

English

Original Letter (Doctor) – Case 10:

Dear Professor Buchfelder,

We are reporting on our mutual patient, Ms. **** ****, who presented herself on 18.07.2018 along with her two daughters at our radiation therapy outpatient clinic.

At your clinic on 29.06.2018, a right frontal tumor resection was performed. Histopathologically, it was identified as glioblastoma multiforme, WHO Grade IV.
There is now an indication for postoperative simultaneous radiochemotherapy.

We have thoroughly informed and advised the patient about the planned radiotherapy, and she consented to the treatment.
At the conclusion of the consultation, we coordinated the following appointments:

- Mask Fabrication: 26.07.2018, 09:00 AM
- MRI of the Skull: 26.07.2018, 19:30 PM
- Day Clinic: 30.07.2018, 11:00 AM

You will, of course, receive a detailed report on the further progress.
We thank you for the collegial cooperation and remain

with kind collegial regards,

Prof. Dr. R. Fietkau
Director of the clinic

Dr. G. Lahmer
Senior Physician of the Clinic

#	**Make a mark for each category!**	✘

Comparison of AI letter with the original letter:

1	The original letter is better	
2	The original letter and the AI letter are equally good	
3	The AI letter is better	

For the generation of the **summary letters**, the patient's diagnosis and oncologic history were used as input and the summary letter was used as a label for LLM fine-tuning. Here is a **Translated example:**

{"input": "Diagnosis Diagnosis: Glioblastoma left temporo-occipital, IDH-1 wild type, WHO grade IV, MGMT promoter methylation Secondary diagnoses: Art. Hypertension Pollen allergy Tumor-specific anamnesis ##.##.####: Left temporo-parietal, basal craniotomy Tumor resection (Neurosurgical Clinic, University of Erlangen) Histology (Neuropathological Institute, University of Erlangen): Glioblastoma, IDH-1 wild type, WHO grade IV. ##.##.####: MRI skull assessment: No evidence of high-grade tumor residue/recurrence after extirpation of a glioblastoma multiforme WHO grade IV temporoparietal left on December 2nd ####. Postoperative changes, or low-grade tumor parts adjacent to the resection defect. ##.##.#### - ##.##.####: Postoperative simultaneous Radiochemotherapy Radiotherapy in VMAT Single dose 2 Gy, total dose 60 Gy Chemotherapy CeCeNu 100 mg/m^2 BSA d 1, 200 mg absolute Temodal 100 mg/m^2 BSA d 2-6, 200 mg absolute WH d 42 to #/#### Maintenance chemotherapy with CCNU/Temodal (total of 6 cycles, no Temodal in cycle 6) [...]

"output": "The patient was diagnosed with a left temporoparietal glioblastoma, WHO Grade IV, with IDH-1 wild type and MGMT promoter methylation. On December #, ####, he underwent a basal craniotomy for tumor resection, which confirmed the diagnosis of glioblastoma. Subsequent MRI on January ##, ####, showed no evidence of higher-grade residual tumor after the resection, although there were postoperative changes and low-grade tumor remnants adjacent to the resection defect.\n\nFollowing the surgery, the patient received concurrent radiochemotherapy from January ##, ####, to March #, ####, with radiotherapy (60 Gy) and chemotherapy (CeCeNu and Temodal). He is currently undergoing maintenance chemotherapy with CCNU/Temodal, with the next follow-up and interdisciplinary tumor center coordination planned according to the guidelines for radiation protection in medicine. [...]" },
